# Ghrelin-responsive mediobasal hypothalamic neurons mediate exercise-associated food intake and exercise endurance

**DOI:** 10.1172/jci.insight.172549

**Published:** 2023-12-22

**Authors:** Omprakash Singh, Sean B. Ogden, Salil Varshney, Kripa Shankar, Deepali Gupta, Subhojit Paul, Sherri Osborne-Lawrence, Corine P. Richard, Nathan P. Metzger, Connor Lawrence, Luis Leon Mercado, Jeffrey M. Zigman

**Affiliations:** 1 Center for Hypothalamic Research, Department of Internal Medicine;; 2 Division of Endocrinology & Metabolism, Department of Internal Medicine; and; 3 Department of Psychiatry, University of Texas Southwestern Medical Center, Dallas, Texas, USA.

**Keywords:** Metabolism, Neuroscience, G protein–coupled receptors

## Abstract

Previous studies have implicated the orexigenic hormone ghrelin as a mediator of exercise endurance and the feeding response postexercise. Specifically, plasma ghrelin levels nearly double in mice when they are subjected to an hour-long bout of high-intensity interval exercise (HIIE) using treadmills. Also, growth hormone secretagogue receptor–null (GHSR-null) mice exhibit decreased food intake following HIIE and diminished running distance (time until exhaustion) during a longer, stepwise exercise endurance protocol. To investigate whether ghrelin-responsive mediobasal hypothalamus (MBH) neurons mediate these effects, we stereotaxically delivered the inhibitory designer receptor exclusively activated by designer drugs virus AAV2-hSyn-DIO-hM4(Gi)-mCherry to the MBH of *Ghsr-IRES-Cre* mice, which express Cre recombinase directed by the *Ghsr* promoter. We found that chemogenetic inhibition of GHSR-expressing MBH neurons (upon delivery of clozapine-*N*-oxide) 1) suppressed food intake following HIIE, 2) reduced maximum running distance and raised blood glucose and blood lactate levels during an exercise endurance protocol, 3) reduced food intake following ghrelin administration, and 4) did not affect glucose tolerance. Further, HIIE increased MBH *Ghsr* expression. These results indicate that activation of ghrelin-responsive MBH neurons is required for the normal feeding response to HIIE and the usual amount of running exhibited during an exercise endurance protocol.

## Graphical abstract

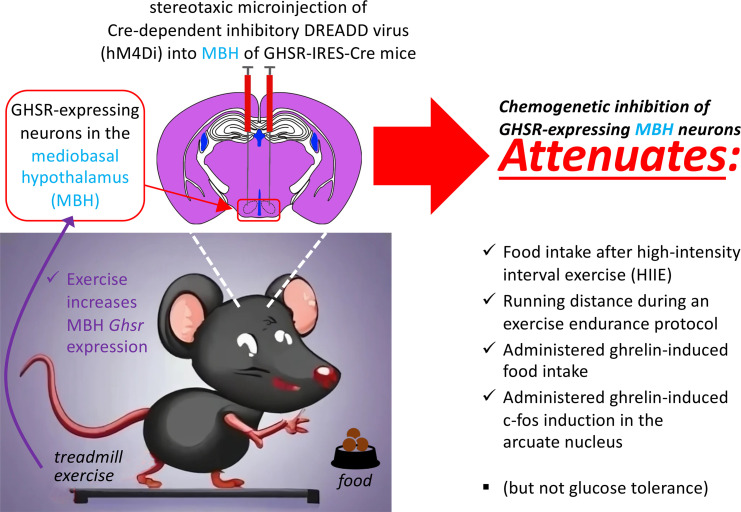

## Introduction

The mainly stomach-derived, acylated hormone ghrelin acts via growth hormone secretagogue receptors (GHSRs) to regulate food intake, blood glucose, and muscle function (1). Regarding food intake, ghrelin is orexigenic, potently stimulating eating when administered, ensuring appropriate rebound hyperphagia in response to short-term fasting, and engaging hedonic eating behaviors, for instance, in response to chronic psychosocial stress (2–4). Regarding blood glucose, administered ghrelin increases blood glucose while ghrelin deletion and GHSR deletion lead to progressive declines in blood glucose into the hypoglycemic range over a weeklong severe caloric restriction regimen (5–7). Regarding muscle function, administering ghrelin, GHSR agonists, or an agent that boosts plasma ghrelin ameliorates reduced muscle contraction force and skeletal muscle wasting in rodent cachexia and muscle atrophy models and improves muscle strength in patients with cancer cachexia (8–13).

These actions of the ghrelin system are highlighted by exercise. For example, in a mouse chronic kidney disease model, ghrelin administration increases exercise endurance, gastrocnemius mass, and gastrocnemius muscle fiber size (13). As we have reported, an hour-long bout of forced high-intensity interval exercise (HIIE) using treadmills nearly doubles plasma ghrelin levels in mice (14). This effect lasts at most 0.5–2 hours (14). The effect of exercise on plasma ghrelin has also been investigated in humans using exercise regimens such as treadmills, cycling, and rowing; some studies report high plasma ghrelin and others report lower or unchanged ghrelin levels, as discussed previously (14). Also, whereas HIIE does not acutely increase food intake in wild-type mice over that observed in sedentary wild-type mice, GHSR-null mice exhibit marked reductions in food intake (by ~70%) in the first 4 hours following HIIE as compared with exercised wild-type littermates (14). Further, although both GHSR-null mice and wild-type littermates are able to complete the HIIE protocol, when submitted to a longer, stepwise exercise endurance protocol, GHSR-null mice exhibit diminished endurance, reaching exhaustion after having run far less distance (by ~30%) and for far less time (by ~20%) than wild-type littermates (14). Moreover, plasma ghrelin levels measured at the time of exhaustion are positively correlated with distance run (14). These data suggest that exercise-induced increases in plasma ghrelin limit the capacity of exercise to restrict food intake following exercise, though they enhance exercise endurance.

Where ghrelin acts to have these effects on food intake following exercise and on exercise endurance is unclear. However, lines of evidence suggest the mediobasal hypothalamus (MBH) is involved. For instance, the ventromedial hypothalamus (VMH) and the arcuate hypothalamus (ARC), which comprise the MBH, regulate some metabolic responses to exercise (see discussion below) (15–19). Further, these MBH nuclei, and closely adjacent hypothalamic nuclei including the dorsomedial hypothalamus (DMH) and premammilary nucleus, ventral part (PMV), represent key sites of GHSR expression and ghrelin action (7, 20–25). Here, we aimed to determine whether GHSR-expressing neurons in the MBH mediate effects of exercise on food intake and regulate exercise endurance.

## Results

### Confirmation of expected Cre recombinase activity within GHSR-expressing neurons of Ghsr-IRES-Cre mice.

**Figure 1:**
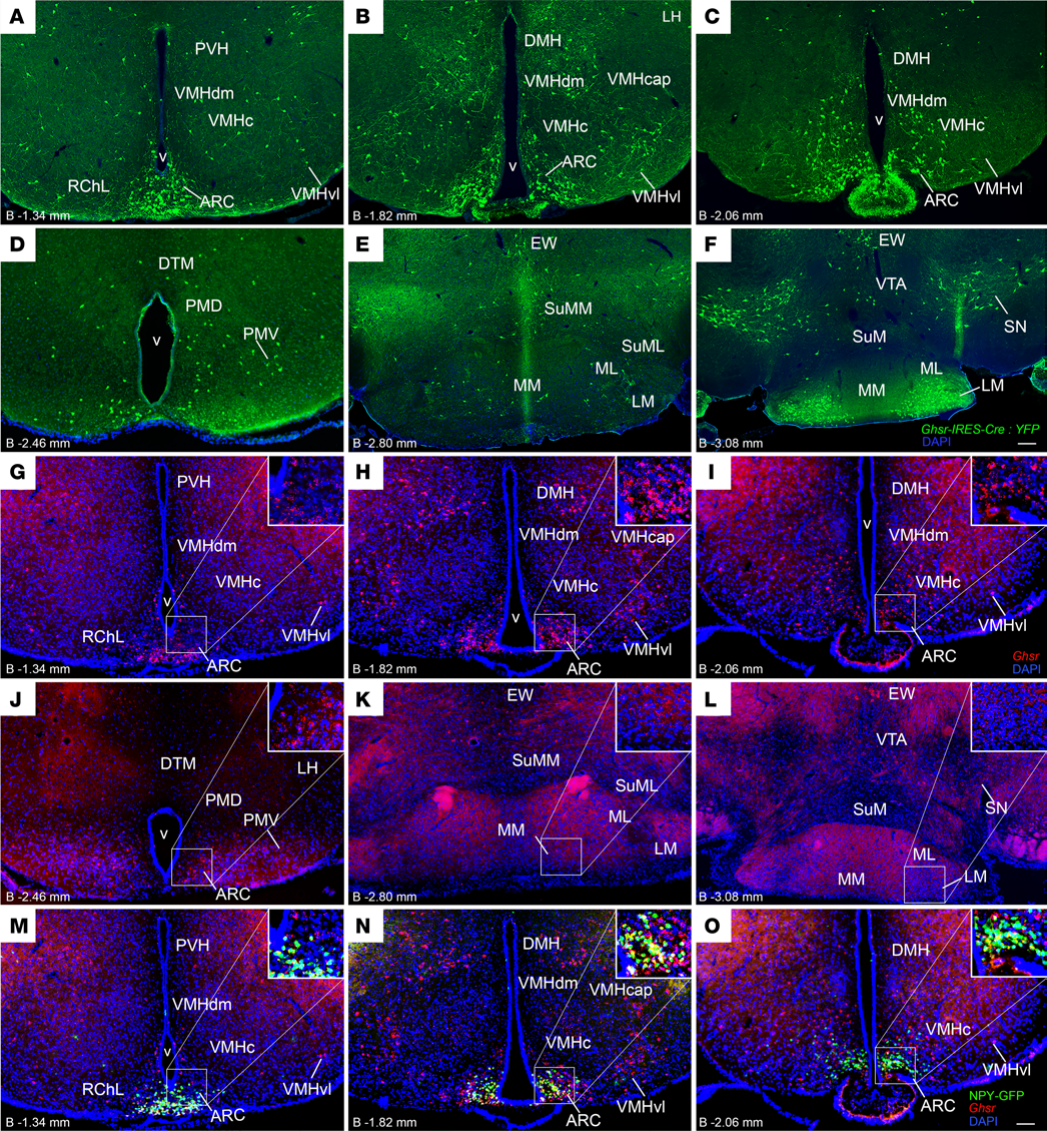
Verification of expected Cre recombinase activity within GHSR-expressing neurons of *Ghsr-IRES-Cre* mice. (**A**–**F**) Low-magnification fluorescence photomicrographs showing YFP-immunoreactive cell bodies (green) in coronal MBH and midbrain sections of a representative *Ghsr-IRES-Cre* ROSA26-YFP reporter mouse. (**G**–**L**) Low-magnification fluorescence photomicrographs showing *Ghsr* mRNA expression (red), as determined using RNAscope in situ hybridization histochemistry in coronal MBH and midbrain sections of a representative NPY-GFP mouse. (**M**–**O**) Low-magnification fluorescence photomicrographs showing expression of *Ghsr* mRNA (red), GFP (green), and their colocalization (yellow) in coronal MBH sections of a representative NPY-GFP mouse. Scale bars = 100 μm in **A**–**O**. Approximate distance of each coronal section from bregma (“B”) is indicated in the lower left corner of each panel. v, third ventricle; ARC, arcuate nucleus; DMH, dorsomedial hypothalamic nucleus; DTM, dorsal tuberomammillary nucleus; EW, Edinger-Westphal nucleus; LM, lateral mammillary nucleus; MM, medial mammillary nucleus, medial part; ML, medial mammillary nucleus, lateral part; PMD, premammillary nucleus, dorsal part; PMV, premammillary nucleus, ventral part; PVH, paraventricular hypothalamic nucleus; RChL, retrochiasmatic area, lateral part; SN, substantia nigra; SuML, supramammillary nucleus, lateral part; SuMM, supramammillary nucleus, medial part; VMHc, ventromedial hypothalamic nucleus, central aspect; VMHcap, ventromedial hypothalamic nucleus, capsular region; VMHdm, ventromedial hypothalamic nucleus, dorsomedial aspect; VMHvl, ventromedial hypothalamic nucleus, ventrolateral aspect; VTA, ventral tegmental area.

We began by verifying selectivity of Cre recombinase activity to GHSR-expressing cells of a previously reported *Ghsr-IRES-Cre*–knockin mouse line (21). This was achieved by crossing *Ghsr-IRES-Cre* mice to Cre-dependent ROSA26-YFP reporter mice and examining YFP expression within 10- to 12-week-old progeny containing 1 *Ghsr-IRES-Cre* allele and 1 ROSA26-YFP transgene. YFP expression within coronal brain sections extending approximately –1.34 mm to –3.08 mm from bregma (which spans the MBH to the midbrain) was compared with *Ghsr* mRNA expression as determined by in situ hybridization histochemistry (ISHH) using RNAscope in corresponding sections from NPY-hrGFP mice (26). Cre activity, as indicated by YFP reporter expression (Figure 1, A–F), matched that of *Ghsr* mRNA expression (Figure 1, G–L). As reported previously (20, 21, 27), regions containing GHSR-expressing cells included the ARC, VMH, DMH, PVH, LH, PMV, PMD, DTM, LM, MM, ML, VTA, and SN. Isolated GHSR-expressing cells also were observed in the SuML, SuMM, and EW.

Regarding the ARC, *Ghsr* mRNA expression was highly localized to NPY neurons, as indicated by colocalization with GFP (Figure 1, M–O). Regarding the VMH, both Cre activity and *Ghsr* mRNA expression were observed sparsely in the dorsomedial (VMHdm) and central (VMHc) aspects and more prominently in the ventrolateral (VMHvl) aspect and its capsule (VMHcap), as had been described earlier for Cre activity (21). Notably, ISHH studies using a ^35^S-labeled antisense GHSR riboprobe previously had demonstrated *Ghsr* mRNA expression in mouse VMH to be restricted to the VMHvl and VMHcap (20), although GHSR expression in the VMHdm and VMHc is well established in the rat (20, 28). VMHvl expression of *Ghsr* mRNA within the mouse also has been established by RNA sequencing (29).

Additionally, although not done in our previous studies utilizing the *Ghsr*-*IRES-Cre* line (21, 30, 31), we performed dual-label histochemistry using MBH coronal sections to determine the overlap of Cre-dependent YFP immunoreactivity in the *Ghsr*-*IRES-Cre* ROSA26-YFP reporter mouse line with *Ghsr* mRNA expression, as determined using RNAscope ISHH. As indicated in Supplemental Figure 1, A–D (supplemental material available online with this article; https://doi.org/10.1172/jci.insight.172549DS1), 100% of YFP-expressing cells coexpressed *Ghsr* (as indicated by the overlapping red fluorescence RNAscope signal, which represents antisense *Ghsr* riboprobes). The same coexpression pattern was observed in a second representative mouse at 2 slightly more caudal levels (Supplemental Figure 1, E–H and K–N). Notably, single-label YFP immunohistochemistry and single-label *Ghsr* RNAscope ISHH were also performed on 2 adjacent sections (see Supplemental Figure 1, I and J, respectively), both of which were adjacent to the section that underwent dual-label histochemistry (Supplemental Figure 1, E–H). A reduced number of YFP-immunoreactive cells was observed in the dual-labeled section than the section that underwent single-label YFP immunohistochemistry, illustrating a known caveat of dual-label histochemistry to reduce the labeling of 1 or both signals.

Although 100% of YFP-expressing cells coexpressed the red fluorescence RNAscope signal, the RNAscope signal was also observed without colocalized YFP signal (Supplemental Figure 1, D, H, and N). In other words, Cre activity was localized only to *Ghsr*-expressing cells, but not all *Ghsr*-expressing cells expressed Cre activity. While it is not uncommon for Cre activity to be underrepresented in target cells (in this case, *Ghsr*-expressing cells), 2 known caveats of the *Ghsr*-*IRES-Cre* mouse might be impacting the histochemistry results. First, as originally reported (21), although we often observe a pattern of Cre activity in *Ghsr*-*IRES-Cre* mice that matches the known pattern of *Ghsr* mRNA expression, occasional *Ghsr*-*IRES-Cre* reporter mice have exhibited a somewhat asymmetric pattern (more expression on one side of the brain) or a less extensive bilateral pattern of Cre activity (in which Cre activity is missing from some of the usual sites of *Ghsr* expression). The reasons for these alternate patterns of Cre activity in a subset of the reporter mice are unclear. Also, the original characterization of the *Ghsr*-*IRES-Cre* line demonstrated some slight differences in numbers of observed cells containing Cre activity within certain brain regions, which seemed dependent on the reporter line used (21). Second, in a follow-up study, mice heterozygous for the *Ghsr*-*IRES-Cre* allele (as are the mice used here) exhibited a 34% reduction in the number of *Ghsr* mRNA–expressing cells within the ARC compared with wild-type littermates (31). That said, food intake and ARC c-Fos induction in response to administered ghrelin were similar to those of wild-type mice, suggesting the slight reduction in *Ghsr* expression in *Ghsr*-*IRES-Cre* mice did not affect responsiveness to ghrelin (31). Both of these caveats, together with reductions in observed signal that occur as a result of dual-label staining (see above), likely affect the observed degree of colocalization of Cre-driven YFP signal within cells containing *Ghsr* mRNA–associated red fluorescence.

### HIIE increases GHSR expression in the MBH.

**Figure 2:**
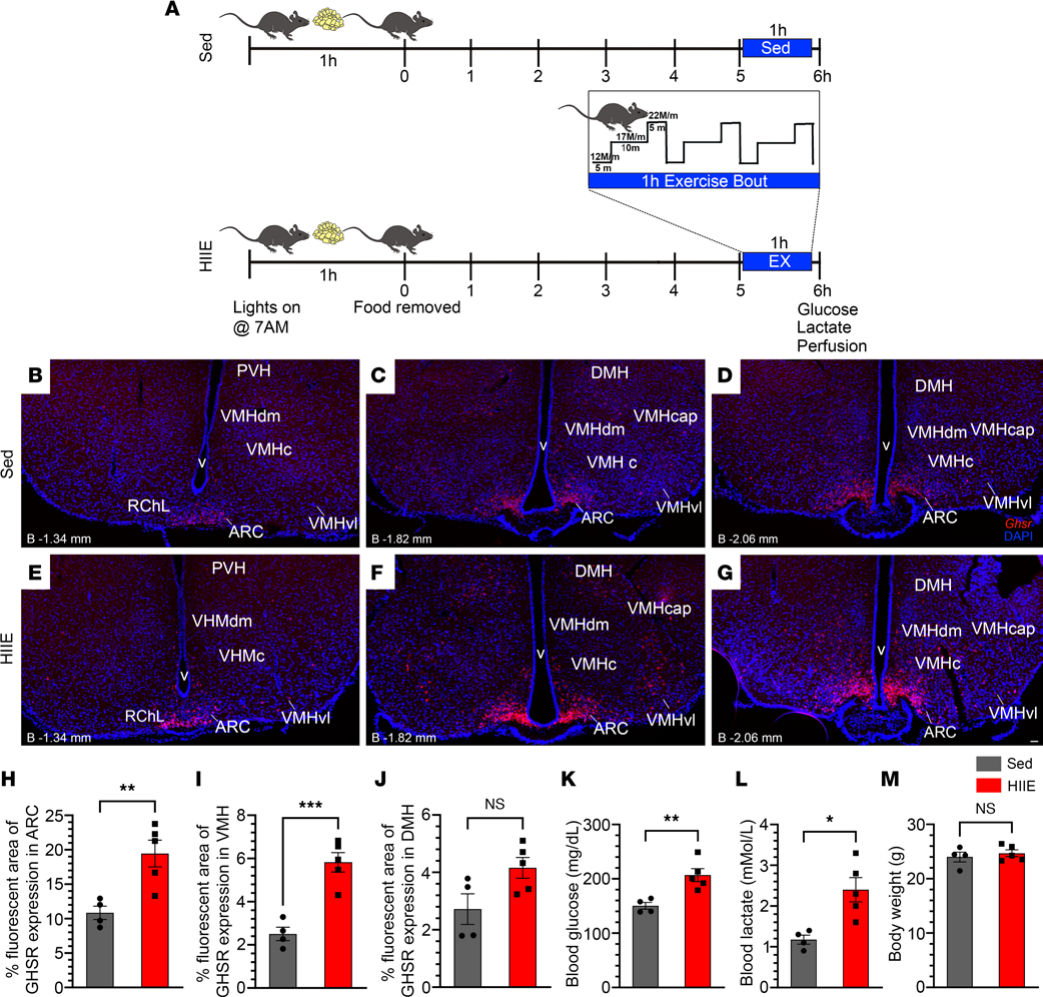
HIIE increases hypothalamic *Ghsr* expression. (**A**) Schematic of the experimental design. (**B**–**G**) Low-magnification fluorescence photomicrographs showing *Ghsr* mRNA expression (red), as determined using RNAscope in situ hybridization histochemistry in coronal MBH sections of a representative sedentary (sed) mouse (**B**–**D**) and a representative mouse that underwent HIIE (**E**–**G**). DAPI (blue) is used as counterstaining. Approximate distances of each coronal section from bregma (“B”) are indicated in the lower left corner of each panel. Scale bars = 50 μm in **B**–**G**. (**H**–**J**) Effects of HIIE on the percentage fluorescent area representing *Ghsr* expression within the (**H**) ARC, (**I**) VMH, and (**J**) DMH. (**K** and **L**) Effects of HIIE on blood (**K**) glucose and (**L**) lactate. (**M**) Body weights of mice (sedentary mice, *n* = 4; versus mice that underwent HIIE, *n* = 5). Unpaired Student’s *t* test (2 tailed). *P < 0.05, **P < 0.01, ***P < 0.001.

To determine the effects of exercise on central GHSR expression, we subjected 10- to 12-week-old C57BL/6N to HIIE. Mice were familiarized to treadmills over 2 successive days and then were submitted to an HIIE protocol the next day. This consisted of withdrawing food 1 hour after lights-on, then 5 hours later, submitting mice to the 1-hour HIIE bout, followed within 5 minutes by a tail nick to sample blood for glucose and lactate measurements and then immediate anesthetization and transcardial perfusion; sedentary control mice were treated similarly except they were kept sedentary during the period the others underwent HIIE (Figure 2A). As compared with sedentary control mice (Figure 2, B–D, and Supplemental Figure 2, A–D), the exercised mice (Figure 2, E–G, and Supplemental Figure 2, E–I) exhibited higher MBH expression of *Ghsr* mRNA, as determined using RNAscope ISHH. These changes were observed at all 3 levels of the MBH that were examined, including coronal sections located –1.34, –1.82, and –2.06 mm from bregma, within the ARC and VMH, but not the DMH (Figure 2, H–J). Quantification demonstrated that HIIE was associated with 79.4% and 132% increases in percentage fluorescent area (representing *Ghsr* expression) in the ARC and VMH, respectively.

Also, we verified the previous observation that HIIE acutely raised blood glucose (by 37.6% as compared with sedentary mice; Figure 2K) (14). For the first time to our knowledge, we show that HIIE also acutely raised blood lactate (by 104.3%; Figure 2L). HIIE did not affect body weight (Figure 2M).

### Inhibition of GHSR-expressing MBH neurons attenuates food intake after HIIE.

**Figure 3:**
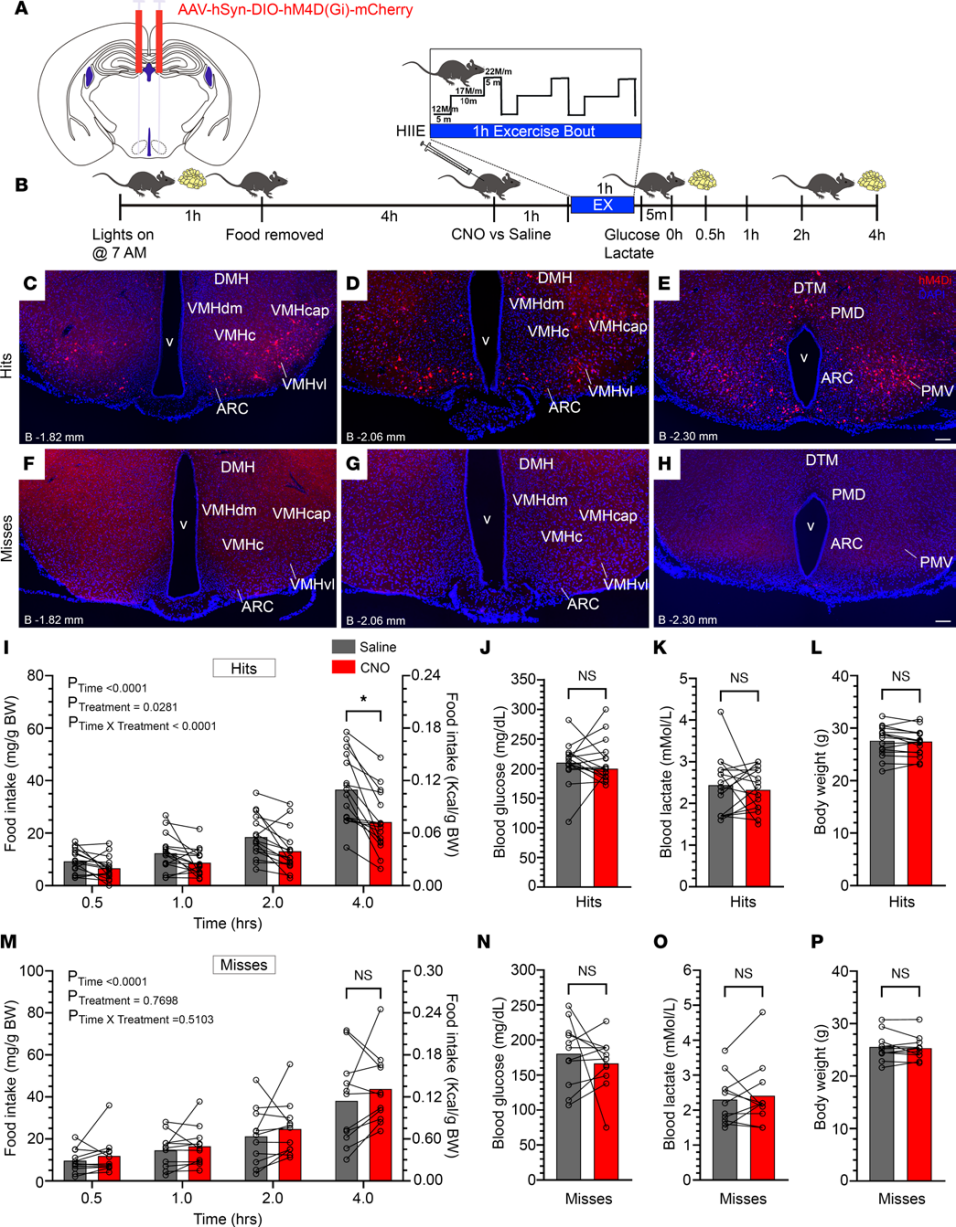
Inhibition of GHSR-expressing MBH neurons attenuates food intake after HIIE. (**A**) Schematic of coronal brain section demonstrates the sites of injection of AAV-hSyn-DIO-hM4D(Gi)-mCherry virus (hM4Di) within the MBH of *Ghsr-IRES-Cre* mice. (**B**) Schematic of the experimental design. (**C**–**H**) Representative coronal brain sections from *Ghsr-IRES-Cre* mice injected with the virus, demonstrating Cre-dependent mCherry expression in the MBH (red) or the lack thereof. DAPI (blue) is used as counterstaining. Approximate distances of each coronal section from bregma (“B”) are indicated in the lower left corner of each panel. (**C**–**E**) The top row displays brain sections from a representative mouse classified as a “hit” as a result of a correctly targeted MBH. (**F**–**H**) The bottom row displays brain sections from a representative mouse classified as a “miss” as a result of incorrect targeting of the virus. Scale bar = 100 μm in **C**–**H**. (**I**–**L**) Effects of administration of CNO (0.3 mg/kg BW, i.p.) versus saline in “hits” on (**I**) food intake, (**J**) blood glucose, (**K**) blood lactate, and (**L**) and body weight. (**M**–**P**) Effects of administration of CNO (0.3 mg/kg BW, i.p.) versus saline in “misses” on (**M**) food intake, (**N**) blood glucose, (**O**) blood lactate, and (**P**) and body weight. *n* = 16 “hits” and *n* = 11 “misses.” (**I** and **M**) Repeated measures 2-way ANOVA followed by Holm-Šidák post hoc multiple comparisons test. (**J**–**L** and **N**–**P**) Paired Student’s *t* test (2 tailed). *P < 0.05.

We examined the functional significance of GHSR-expressing MBH neurons in mediating exercise-associated metabolic processes by first determining if inhibiting their activity, as achieved using a Cre-dependent chemogenetic system, reduces food intake after HIIE, similar to what is observed in GHSR-null mice (14). Stereotaxic surgery was used to deliver an inhibitory designer receptor exclusively activated by designer drugs (DREADD) virus [AAV2-hSyn-DIO-hM4(Gi)-mCherry; hM4Di] bilaterally to the MBH of 9- to 11-week-old *Ghsr-IRES-Cre*, using coordinates that were chosen to target the ARC and adjacent nuclei, as had been achieved previously (21) (Figure 3A). Cre-expressing neurons infected with hM4Di express a designer receptor that engages downstream Gi-coupled signaling cascades, which in turn inhibit the activity of those neurons upon pharmacological engagement by clozapine-*N*-oxide (CNO); they also coexpress an mCherry reporter that permits their identification (32, 33). At 3 weeks following stereotaxic injection of hM4Di, mice were familiarized to treadmills over 2 successive days and then were subjected to an HIIE protocol the next day. This consisted of withdrawing food 1 hour after lights-on, delivering CNO (0.3 mg/kg BW i.p.) or saline 4 hours later, and submitting the mice to the 1-hour HIIE bout 1 hour after CNO or saline, followed within 5 minutes by a tail nick to sample blood for glucose and lactate measurements and reintroduction of standard chow diet to assess food intake over the next 4 hours (Figure 3B). One week later, this protocol was repeated on the same mice using a crossover design to deliver saline or CNO.

Following subsequent exercise endurance and administered ghrelin-induced food intake and c-Fos studies (see below), mCherry expression was determined to classify those cases with correctly targeted virus injections as “hits” or mistargeted virus injections as “misses.” “Hits” were defined here as cases with mCherry expression bilaterally or unilaterally in both the ARC and VMH but without mCherry expression caudal to the ARC (e.g., caudal to a distance –2.46 mm from bregma); “hits” most often also expressed mCherry in the DMH, median tuberal nucleus (MTu), PMV, and/or the posterior hypothalamic area (PH). “Misses” were defined here as cases without mCherry expression in both the ARC and VMH; “misses” most often expressed mCherry caudal to a distance –2.46 mm from bregma, often also expressed mCherry in the PMV, and occasionally included cases with minimal mCherry expression in either the ARC or VMH (but not both). mCherry expression within 3 levels of the MBH of a representative “hit” is depicted in Figure 3, C–E, whereas the lack of mCherry expression in the corresponding MBH levels of a representative “miss” is depicted in Figure 3, F–H. A more complete survey of mCherry expression within 5 coronal brain section levels (extending from a distance of –1.34 mm to –2.80 mm from bregma) is included in Supplemental Figure 3 for all 16 “hits,” in Supplemental Figure 4 for all 11 “misses,” and in Table 1. Of those 16 “hits,” 13 showed bilateral targeting whereas 3 (Supplemental Figure 3, M, O, and P) showed unilateral targeting.

Food intake over the first 4 hours after HIIE was significantly attenuated by CNO treatment when compared with saline treatment in the “hits,” with the difference between CNO and saline treatment becoming most prominent at 4 hours (31.3% reduction; Figure 3I). Notably, separate analysis of only those 13 (out of 16) “hits” targeted bilaterally showed similar results; namely, CNO treatment reduced food intake after HIIE by 31.8% (*P* < 0.01) compared with saline (data not shown). CNO treatment did not reduce or otherwise affect exercise-induced food intake in the “misses” (Figure 3M). Neither blood glucose, blood lactate, nor body weight was affected by CNO delivery, in “hits” or “misses” (Figure 3, J–L and N–P).

### Inhibition of GHSR-expressing MBH neurons impairs exercise endurance and MBH c-Fos induction resulting from exercise.

**Figure 4:**
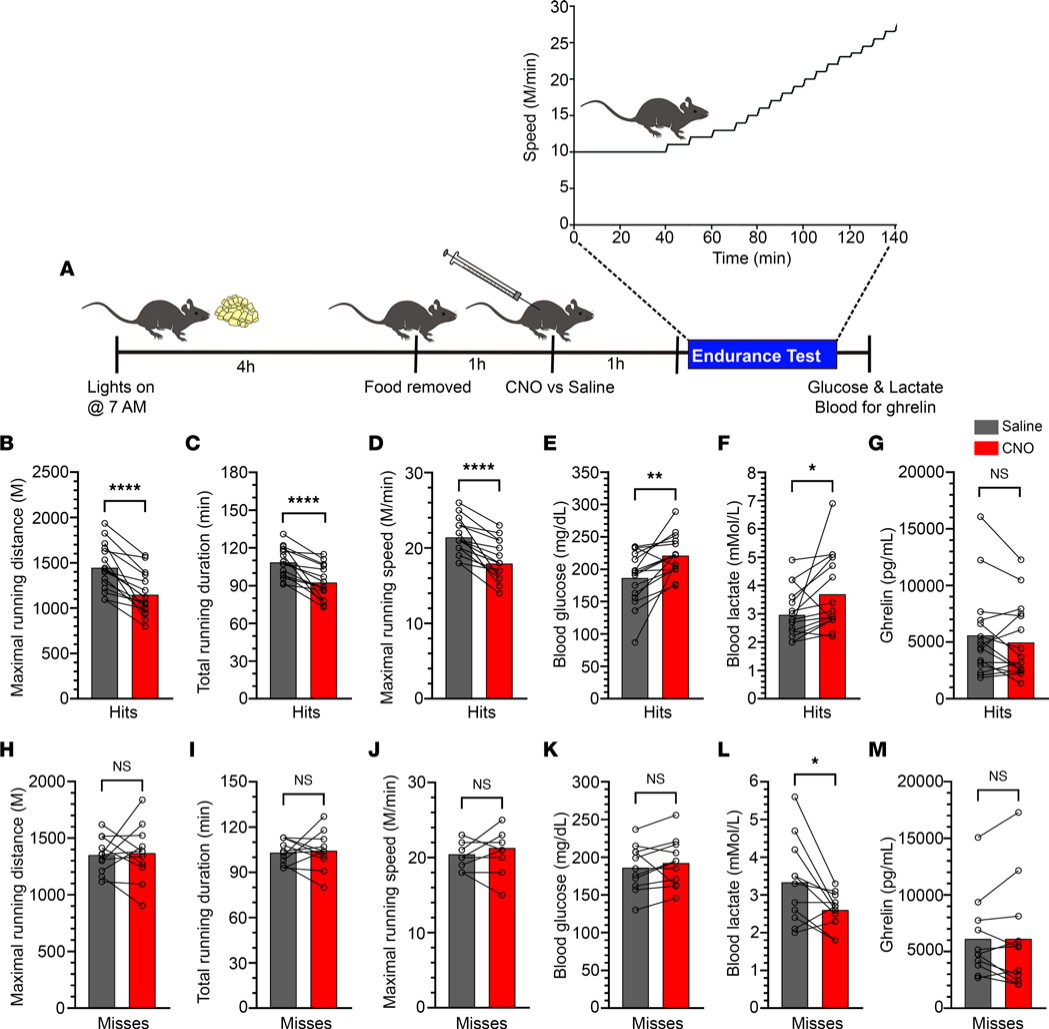
Inhibition of GHSR-expressing MBH neurons impairs exercise endurance. (**A**) Schematic of the experimental design. (**B**–**G**) Effects of administration of CNO (0.3 mg/kg BW, i.p.) versus saline in hM4Di-injected “hits” on (**B**) maximal running distance, (**C**) total running duration, (**D**) maximal running speed, (**E**) blood glucose at exhaustion, (**F**) blood lactate at exhaustion, and (**G**) plasma ghrelin at exhaustion. (**H**–**M**) Effects of administration of CNO (0.3 mg/kg BW, i.p.) versus saline in hM4Di-injected “misses” on (**H**) maximal running distance, (**I**) total running duration, (**J**) maximal running speed, (**K**) blood glucose at exhaustion, (**L**) blood lactate at exhaustion, and (**M**) plasma ghrelin at exhaustion. *n* = 16 “hits” and *n* = 11 “misses.” Paired Student’s *t* test (2 tailed). *P < 0.05, **P < 0.01, ****P < 0.0001.

Next, we determined if chemogenetic inhibition of GHSR-expressing MBH neurons reduces exercise endurance, similar to what is observed in GHSR-null mice (14). One week following the above-described set of HIIE studies in hM4Di-injected mice (Figure 3), access to food was restricted beginning 4 hours after lights-on, CNO (0.3 mg/kg BW i.p.) or saline was delivered 1 hour later, and mice were subjected to a stepwise exercise endurance protocol (lasting ~130 minutes, at most) 1 hour following CNO or saline, after which tails were nicked to obtain blood for glucose, lactate, and ghrelin measurements (Figure 4A). One week later, this protocol was repeated on the same mice using a crossover design to deliver saline or CNO.

Exercise endurance was significantly attenuated by CNO treatment when compared with saline treatment in the “hits,” as evidenced by the CNO-treated “hits” reaching exhaustion after having run only 79.3% as far (Figure 4B) and 85.3% as long (Figure 4C) as saline-treated “hits.” Notably, separate analysis of only those 13 (out of 16) “hits” targeted bilaterally showed similar results; namely, CNO-treated bilateral “hits” reached exhaustion after having run only 77.2% as far (*P* < 0.0001; data not shown). Further, CNO-treated “hits” achieved maximal running speeds that were only 83.8% as fast (Figure 4D) as saline-treated “hits.” Additionally, blood glucose was higher by 18.4% (Figure 4E), and blood lactate was higher by 24.6% (Figure 4F) at exhaustion as a result of CNO treatment in “hits.” In the “misses,” CNO treatment did not affect maximal running distance, total running duration, maximal running speed, or blood glucose at exhaustion (Figure 4, H–L), though it reduced blood lactate at exhaustion (by 22.1%; Figure 4L). Although we have no explanation for the CNO-associated lactate reduction in “misses,” it did not affect their exercise endurance. CNO treatment did not affect plasma ghrelin at exhaustion in “hits” or “misses” (Figure 4, G and M).

**Figure 5:**
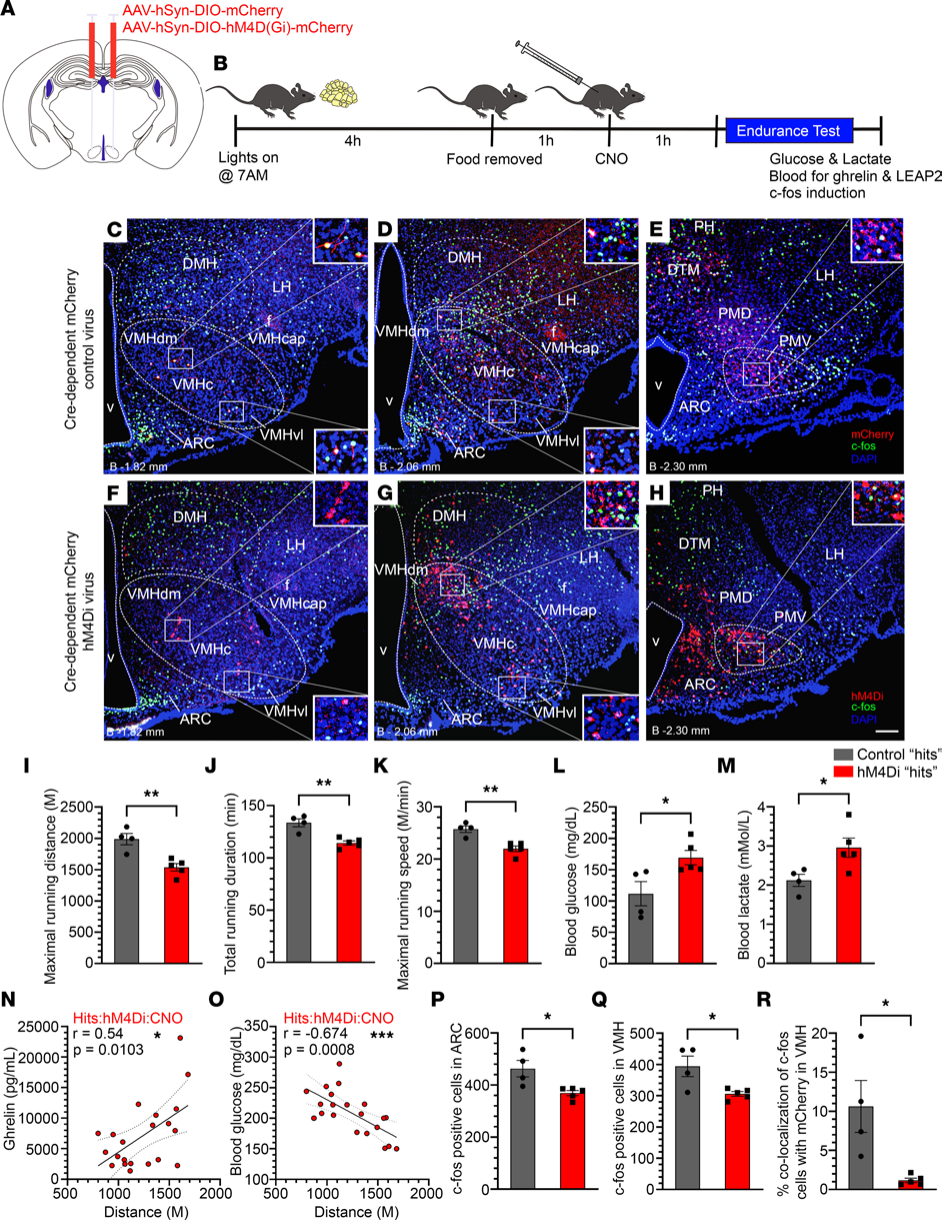
Inhibition of GHSR-expressing MBH neurons impairs exercise endurance and c-Fos induction in the MBH following an exercise endurance test. (**A**) Schematic demonstrating injection sites of AAV-hSyn-DIO-hM4D(Gi)-mCherry (hM4Di) or control AAV-hSyn-DIO-mCherry (“Cre-dependent mCherry control”) viruses. (**B**) Experimental design. (**C**–**H**) Confocal images of coronal brain sections showing c-Fos immunoreactivity (green) and mCherry expression (red) in the MBH and adjacent regions of a representative “Cre-dependent mCherry control virus”–injected “hit” (**C**–**E**) and in a representative hM4Di-injected “hit” (**F**–**H**) sacrificed at exhaustion. DAPI counterstaining is shown in blue. Scale bar in **H** = 100 μm and applies to panels **C**–**H**. Approximate distance from bregma (“B”) is indicated in **C**–**H**. (**I**–**M**) Effects of administration of CNO (0.3 mg/kg BW, i.p.) in hM4Di “hits” (*n* = 5) versus Cre-dependent mCherry control “hits” (*n* = 4) on (**I**) maximal running distance, (**J**) total running duration, (**K**) maximal running speed, (**L**) blood glucose, and (**M**) blood lactate, in exercised mice at exhaustion. (**N** and **O**) Correlations between (**N**) plasma ghrelin and (**O**) blood glucose with distance run in CNO-treated hM4Di “hits” (n = 21, including the 5 hM4Di “hits” from this study and the 16 “hits” from Figure 4). (**P**–**R**) Numbers of c-Fos–immunoreactive cells in the (**P**) ARC and (**Q**) VMH of hM4Di “hits” (*n* = 5) versus Cre-dependent mCherry control “hits” (*n* = 4) following CNO and the exercise endurance test. (**R**) Percentage colocalization of c-Fos–positive cells with mCherry in VMH, hM4Di “hits” (*n* = 5) versus Cre-dependent mCherry control “hits” (*n* = 4), following CNO and the exercise endurance test. Data were analyzed by unpaired Student’s *t* test (2 tailed) (**I**–**M** and **P**–**R**) or Pearson’s correlation and simple linear regression analysis (**N** and **O**). Pearson’s correlation coefficient (*r*) and *P* values are indicated in the figure panels. Solid lines represent the fitted linear regression curves and the dotted lines represent the SEM. *P < 0.05, **P < 0.01, ***P < 0.001.

A separate set of exercise endurance studies was performed using a different set of controls. Whereas the above mice were injected with hM4Di and assessed in a crossover fashion following CNO versus saline, here, mice were injected with 1 of 2 viruses (hM4Di or a noninhibitory control virus), and then all were assessed after CNO administration. Specifically, CNO (0.3 mg/kg BW i.p.) was administered to 12- to 14-week-old *Ghsr-IRES-Cre* mice, which had received bilateral MBH stereotaxic injections of either hM4Di or a control AAV-hSyn-DIO-mCherry (“Cre-dependent mCherry control”) virus 4 weeks earlier (Figure 5A). Afterward, the same protocol as described above (Figure 4A) was used, except a) 1 week earlier, the mice were submitted to an oral glucose tolerance test (oGTT) protocol (see below), b) each of the 2 exercise endurance runs was proceeded by CNO (instead of once with CNO and once with saline), c) plasma liver-expressed antimicrobial peptide-2 (LEAP2) at exhaustion also was checked, and d) the mice were anesthetized and transcardially perfused with formalin immediately following the blood collection (Figure 5B). Post hoc immunohistochemical analysis of coronal brain sections identified 4 Cre-dependent mCherry control “hits” and 5 hM4Di “hits” (Figure 5, C–H, and Supplemental Figure 5).

Just as had been observed in hM4Di-injected “hits” after CNO treatment versus saline (Figure 4), CNO treatment reduced maximal running distance (by 22.7%; Figure 5I), total running duration (by 14.5%; Figure 5J), and maximal running speed (by 14.5; Figure 5K) in hM4Di-injected “hits” versus Cre-dependent mCherry control virus–injected “hits.” Also similar to hM4Di-injected “hits” after CNO treatment versus saline (Figure 4), CNO treatment increased blood glucose (by 51.5%; Figure 5L) and blood lactate (by 39.2%; Figure 5M) at exhaustion in hM4Di-injected “hits” versus Cre-dependent mCherry control virus–injected “hits.” Neither plasma ghrelin at exhaustion, plasma LEAP2 at exhaustion, nor the LEAP2/ghrelin molar ratio (which helps determine the degree of ghrelin resistance) (34) at exhaustion was impacted by chemogenetic inhibition of GHSR-expressing MBH neurons (Supplemental Figure 6, A–C). However, when we grouped the data from all hM4Di-injected “hits” after CNO treatment from Figures 4 and 5 and Supplemental Figure 6, we demonstrated that plasma ghrelin positively correlated with distance run (just as had been shown previously in wild-type mice) (14), blood glucose negatively correlated with distance run, and blood lactate did not correlate with distance run (Figure 5, N–O, and Supplemental Figure 6D).

Further, CNO treatment of hM4Di-injected “hits” reduced the amount of neuronal activation in the MBH observed at exhaustion as compared with CNO treatment of Cre-dependent mCherry control virus–injected “hits.” Specifically, the numbers of c-Fos–immunoreactive cells in the ARC and VMH were reduced by 20.3% and 22.4%, respectively, in the hM4Di-injected “hits” versus the control virus–injected “hits” (Figure 5, P and Q). The percentage of c-Fos–immunoreactive cells in the VMH that coexpressed mCherry was reduced by 88.9% in the hM4Di-injected “hits” versus the control virus-injected “hits” (Figure 5R). No differences were observed in c-Fos immunoreactivity within the DMH or PMV (Supplemental Figure 6, E and F).

**Figure 6:**
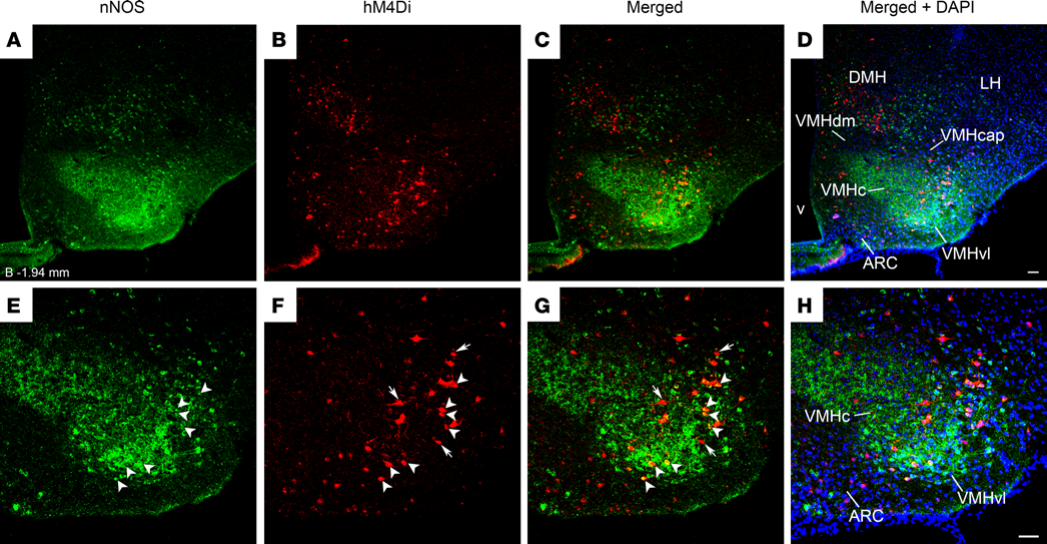
Overlap of nNOS- and Cre-dependent mCherry expression within the MBH of an hM4Di-injected *Ghsr-IRES-Cre* “hit.” (**A**–**D**) Low- and (**E**–**H**) high-magnification confocal images of a coronal brain section (approximately –1.94 mm from bregma) from a representative *Ghsr-IRES-Cre* mouse correctly targeted with an injection of hM4Di into the MBH. This mouse was sacrificed at the time of exhaustion after having received CNO and then being submitted to the exercise endurance protocol schematized in Figure 5B. (**A** and **E**) nNOS immunoreactivity (green). (**B** and **F**) mCherry fluorescence (red). (**C** and **G**) Merged images to demonstrate coexpression of nNOS and mCherry (yellow). (**D** and **H**) Merged images with DAPI counterstain in blue. Arrows point to neurons that exclusively express mCherry (correctly targeted GHSR-expressing neurons). Arrowheads point to neurons that coexpress mCherry + nNOS. *n* = 3 cases analyzed. Scale bar in **D** = 100 μm and applies to **A**–**D**. Scale bar in **H** = 100 μm and applies to **E**–**H**.

To better characterize the chemical phenotypes of the GHSR-expressing neurons of the hM4Di-injected “hits,” we assessed their expression of neuronal nitric oxide synthase (nNOS), which, within the MBH, is most highly expressed in the VMHvl (35). Approximately 63.3% of VMHvl cells expressing mCherry (63.3% of the GHSR-expressing VMHvl neurons inhibited upon CNO administration) coexpressed nNOS immunoreactivity; about 26% of nNOS-immunoreactive cells in the VMHvl coexpressed mCherry (Figure 6).

### Inhibiting GHSR-expressing MBH neurons reduces food intake and MBH c-Fos induction in response to ghrelin.

**Figure 7:**
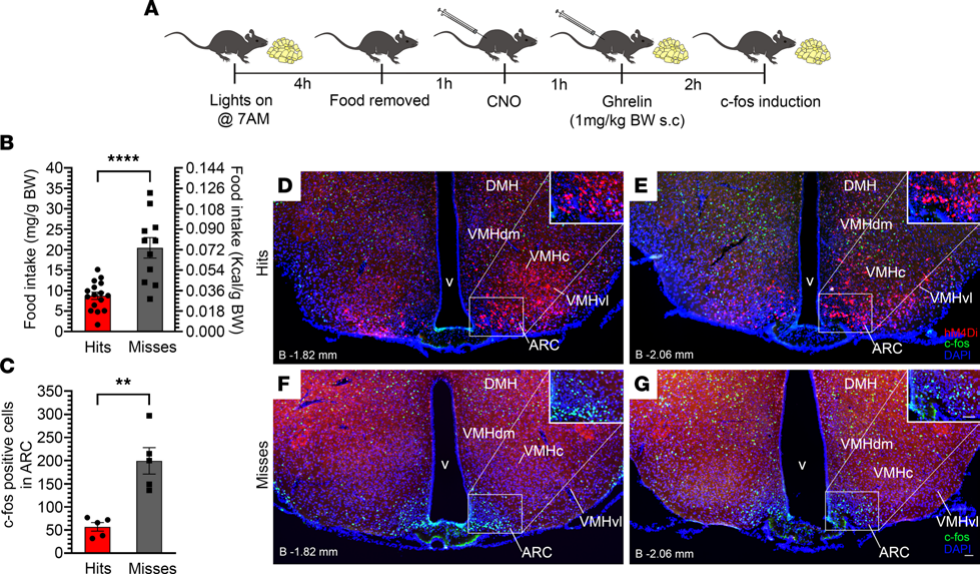
Inhibition of GHSR-expressing MBH neurons reduces food intake and MBH c-Fos induction in response to administered ghrelin. (**A**) Schematic of the experimental design. (**B** and **C**) Effects of administration of CNO (0.3 mg/kg BW, i.p.) in hM4Di-injected *Ghsr-IRES-Cre* “hits” versus “misses” on (**B**) food intake and (**C**) c-Fos induction within the ARC measured 2 hours following delivery of ghrelin (1 mg/kg BW s.c.). (**D**–**G**) Fluorescence images of coronal brain sections showing c-Fos immunoreactivity (green) and mCherry expression (red) in the MBH of a representative hM4Di-injected “hit” (**D** and **E**) and a representative hM4Di-injected “miss” (**F** and **G**) sacrificed 2 hours following ghrelin delivery. DAPI counterstaining is shown in blue. Scale bar in **G** = 50 μm and applies to panels **D**–**G**. Approximate distance of the coronal section from bregma (“B”) is indicated in the lower left corner of panels. *n* = 16 “hits” and *n* = 11 “misses” were used for food intake measurements. *n* = 5 “hits” and *n* = 5 “misses” were used for quantification of c-Fos induction. (**B** and **C**) Unpaired Student’s *t* test (2 tailed). **P < 0.01, ****P < 0.0001.

To demonstrate that the same GHSR-expressing neurons affecting HIIE-induced food intake and exercise endurance are ghrelin responsive, we determined whether their chemogenetic inhibition would also attenuate food intake and ARC c-Fos induction in response to ghrelin administration. Thus, 1 week following the above-described set of exercise endurance studies in hM4Di-injected mice (Figure 4), access to food was restricted beginning 4 hours after lights-on, CNO (0.3 mg/kg BW i.p.) was delivered 1 hour later, ghrelin (1 mg/kg BW s.c.) was administered 1 hour later, and food intake was measured for 2 hours, after which mice were deeply anesthetized and transcardially perfused in preparation for assessment of c-Fos induction (Figure 7A). Just as had previously been observed (21), ghrelin-induced food intake was significantly attenuated (by 57.2%) in “hits” as compared with “misses” following CNO treatment (Figure 7B). This coincided with a 71.4% reduction (Figure 7C) in the number of c-Fos–immunoreactive cells within the ARC of “hits” (Figure 7, D and E) versus “misses” (Figure 7, F and G).

### Inhibiting GHSR-expressing MBH neurons does not affect glucose tolerance.

**Figure 8:**
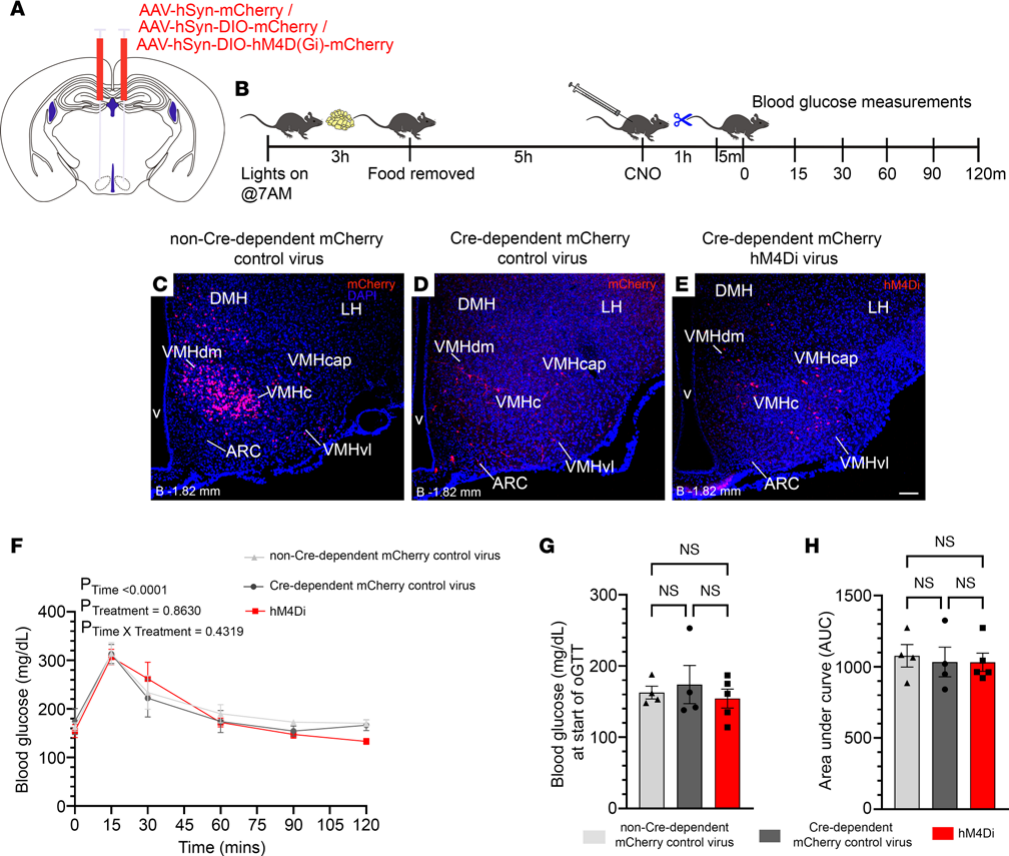
Inhibition of GHSR-expressing MBH neurons does not affect glucose tolerance. (**A**) Schematic of coronal brain section demonstrates the sites of injection of hM4Di, control AAV-hSyn-DIO-mCherry (“Cre-dependent mCherry control”) virus, or AAV-hSyn-mCherry (“non–Cre-dependent mCherry control”) virus within the MBH of *Ghsr-IRES-Cre* mice. (**B**) Schematic of the experimental design. (**C**–**E**) Confocal images of coronal brain sections showing mCherry expression (red) in the MBH of a representative (**C**) “non–Cre-dependent mCherry control virus”–injected “hit,” (**D**) “Cre-dependent mCherry control virus”–injected “hit,” and (**E**) hM4Di-injected “hit.” DAPI counterstaining is shown in blue. Scale bar in **E** = 100 μm and applies to panels **C**–**E**. Approximate distance of the coronal section from bregma (“B”) is indicated in the lower left corner of panels **C**–**E**. (**F**–**H**) Effects of administration of CNO (0.3 mg/kg BW, i.p.) in “non–Cre-dependent mCherry control virus”–injected “hits” (*n* = 4), “Cre-dependent mCherry control virus”–injected “hits” (*n* = 4) and hM4Di-injected “hits” (*n* = 5) on measurements obtained as part of an oGTT: (**F**) blood glucose curves assessed over the first 120 minutes following administration of glucose (2 mg/kg BW) by oral gavage, (**G**) fasting blood glucose levels at the start of the oGTT (after 6-hour fast and just prior to glucose administration), and (**H**) blood glucose AUC. (**F**) Repeated measures 2-way ANOVA with Holm-Šidák post hoc multiple comparisons test. (**G** and **H**) One-way ANOVA with Holm-Šidák multiple comparisons test.

Given the increased blood glucose observed in exercised mice with inhibited GHSR-expressing MBH neurons (Figure 4E and Figure 5L), we next determined if inhibition of GHSR-expressing MBH neurons would impact oral glucose tolerance in the sedentary state. These studies were also supported by previous work demonstrating that ghrelin deletion and GHSR deletion lower fasting blood glucose, improve glucose tolerance, enhance insulin sensitivity, and/or increase glucose-stimulated insulin secretion (36–38). Further, chemogenetic inhibition of steroidogenic factor 1–expressing (SF1-expressing) VMH neurons previously had been shown to worsen glucose tolerance (39). For these studies, we used a new set of 12- to 14-week-old *Ghsr-IRES-Cre* mice, which had received bilateral MBH stereotaxic injections of a control AAV-hSyn-mCherry (“non–Cre-dependent mCherry control”) virus (Figure 8A) 3 weeks earlier (*n* = 4 “hits,” Figure 8C) and the same cohort of “Cre-dependent mCherry control virus”–injected (Figure 8D) versus hM4Di-injected *Ghsr-IRES-Cre* mice (Figure 8E) used in Figure 5. As depicted in Figure 8B, food access was restricted 3 hours following lights-on, CNO (0.3 mg/kg BW i.p.) was administered 5 hours afterward, and 1 hour later, 2 mg/kg BW glucose was administered by oral gavage. Blood glucose was assessed 5 minutes prior to and again at 15, 30, 60, 90, and 120 minutes following glucose delivery. Blood glucose curves are indicated in Figure 8F. There were no differences among the groups in blood glucose levels obtained just prior to glucose administration (after 6-hour fast) (Figure 8G) or AUC (Figure 8H).

## Discussion

These studies reveal that HIIE increases *Ghsr* expression in the MBH (by 79.4% in the ARC and 132% in the VMH), similar to the reported HIIE-associated increase in plasma ghrelin (14). These studies also reveal that activating ghrelin-responsive, GHSR-expressing MBH neurons is required for the normal feeding response to HIIE, amount of endurance, and blood glucose and lactate responses mice exhibit during a stepwise exercise endurance protocol. Specifically, DREADD-assisted inhibition of GHSR-expressing MBH neuronal activity suppressed food intake following HIIE (by 31.3%) and maximal running distance (by 20.7%–22.7%), total running duration (by 14.5%–14.7%), and maximum running speed (by 14.5%–16.1%) during the exercise endurance protocol (hM4Di-injected “hits” treated with CNO versus saline; hM4Di-injected, CNO-treated “hits” versus Cre-dependent mCherry control virus–injected, CNO-treated “hits”). The reduced exercise endurance coincided with a 22.4% and 20.3% reduction in the numbers of c-Fos–immunoreactive cells in the VMH and ARC, respectively (hM4Di-injected “hits” versus Cre-dependent mCherry control virus–injected “hits”). Also, DREADD-assisted inhibition of GHSR-expressing MBH neuronal activity increased blood glucose (by 18.4%–51.5%) and blood lactate (by 24.6%–39.2%) following the exercise endurance protocol (hM4Di-injected “hits” treated with CNO versus saline; hM4Di-injected, CNO-treated “hits” versus Cre-dependent mCherry control virus–injected, CNO-treated “hits”). “Hits” with higher plasma ghrelin at exhaustion exhibited greater exercise endurance whereas “hits” with higher blood glucose exhibited lower exercise endurance. hM4Di-injected “hits” treated with CNO also exhibited a 57.2% reduced food intake and 71.4% reduced c-Fos induction within the ARC in response to ghrelin versus “misses” treated with CNO, verifying the neuronal population being interrogated is responsive to ghrelin and verifying previous studies (21). Chemogenetic inhibition of GHSR-expressing MBH neuronal activity did not affect glucose tolerance. Further, approximately 63.3% of hM4Di-infected, GHSR-expressing VMHvl neurons coexpressed nNOS immunoreactivity, suggesting a possible role for this nNOS subpopulation in mediating HIIE-induced food intake, exercise endurance, and administered ghrelin-induced food intake.

As hinted in the Introduction, evidence in the literature had supported a role of MBH neurons — including those in the VMH and the ARC — in ghrelin’s exercise-related effects. VMH-specific deletion of the transcription factor SF1 reduces exercise endurance and impairs exercise-associated mobilization of several species of free fatty acids (16). Deletion of VMH SF1, which usually is induced by prolonged exercise training, blunts fat mass reductions, blood glucose improvements, and energy expenditure increases associated with exercise training (16). VMH SF1 deletion attenuates the usual metabolic responses of skeletal muscle to exercise, including increases in the mass of several skeletal muscles and induction of muscle PGC-1α expression (16). Further, VMH ablation and blockade of VMH neuronal activity, which may inhibit sympathetic outflow, reduce exercise-induced increases in circulating free fatty acids and their usage (17, 18). Regarding a role of the ARC, exercise induces plasticity within ARC neuronal circuits (19). Specifically, whole-cell patch-clamp recordings indicate that ARC agouti-related protein (AgRP) neurons from exercised mice exhibit a hyperpolarized resting membrane potential, less frequent spontaneous excitatory synaptic currents (sEPSCs), more frequent spontaneous inhibitory synaptic currents (sIPSCs), and decreased action potential frequency (19). In contrast, ARC pro-opiomelanocortin–expressing (POMC) neurons from exercised mice exhibit depolarized resting membrane potential, more frequent sEPSCs, and increased action potential frequency (19). Further, in vivo fiber photometry indicates that HIIE decreases whole-cell calcium levels in ARC AgRP neurons and increases those levels in POMC neurons. These data support a model by which exercise leads to a rapid reorganization of synaptic inputs and biophysical properties of ARC neurons. It is notable that ghrelin, the circulating levels of which rise as a result of HIIE and correlate with distance run in the exercise endurance test, also increases the frequency of sIPSCs onto ARC POMC neurons and excitatory currents in AgRP neurons (40, 41).

Prior evidence for direct engagement of MBH neurons by ghrelin is extensive, especially those populating the ARC (1, 42). As a few examples, not only are GHSRs highly expressed within the MBH, as demonstrated previously and verified here, but also administration of ghrelin and GHSR agonists markedly induces c-Fos within ARC AgRP neurons (22, 43–45). Selective GHSR expression in ARC AgRP neurons is sufficient to allow ghrelin to induce food intake and normalizes the relative hypoglycemia observed in fasted GHSR-null mice (7). Conversely, AgRP neuron–selective GHSR deletion and ablation of ARC AgRP neurons abolish ghrelin’s acute orexigenic effects (24, 46). GHSR expression also occurs in the VMH, including SF1 neurons (21, 47). In rats, in which VMH GHSR expression is much more prominent than in mice (see Results), VMH inhibition of AMPK robustly impairs the central orexigenic effect of ghrelin (23). Further, antisense GHSR shRNA-mediated knockdown of GHSR expression in the VMH reduces wheel running activity in ad libitum–fed rats and rats subjected to a restricted feeding schedule, while also attenuating body weight loss otherwise induced by the wheel running activity (48). Rats with GHSR knockdown in the VMH also exhibit delayed onset of the food-anticipatory activity that characteristically occurs prior to food availability under the restricted feeding schedule (48). Interestingly, VMH GHSR knockdown in ad libitum–fed rats increases food intake and body weight gain (48).

Thus, we are reassured by the findings here demonstrating effects of chemogenetic inhibition of GHSR-expressing MBH neurons to reduce eating after HIIE and after administered ghrelin and to reduce exercise endurance. As no cases had hM4Di targeted selectively to just 1 of the regions comprising the MBH, further work is needed to distinguish the roles of GHSR-expressing neurons in the ARC from those in the VMH or from those in 1 of the other MBH-adjacent sites with GHSR-expressing neurons. Additional studies also are needed to confirm if the effects of chemogenetic inhibition are the result of blocking ghrelin action on those neurons as opposed to a more generalized effect on the activity of those neurons unrelated to ghrelin or GHSR constitutive activity (1). Certainly, the fact that chemogenetic inhibition of the GHSR-expressing MBH neurons reproduces the exercise phenotype of GHSR-null mice suggests that ghrelin and GHSR indeed are involved. Yet, studies using Cre-mediated GHSR deletion within the MBH, or within a specific MBH region or specific MBH neuronal subtype, would undoubtedly help facilitate confirmation of that hypothesis.

Although the current study establishes key effects of GHSR-expressing MBH neurons to impact metabolic changes and exercise endurance, it only scratches the surface regarding the downstream mechanisms by which these occur. The observed changes in blood lactate and blood glucose levels are among the clues. In exercise endurance–tested mice, blood lactate levels were higher at the point of exhaustion when GHSR-expressing MBH neurons were chemogenetically inhibited. This finding suggests that activation of these neurons directs lactate utilization, facilitating greater endurance; in contrast, inhibition of these neurons would lead to underutilization of lactate, thereby reducing endurance. Indeed, improved metabolism of lactate is just one of many adaptations that enable endurance athletes to sustain work (49). Blood glucose levels were also higher at the point of exhaustion when GHSR-expressing MBH neurons were chemogenetically inhibited, and there was a negative correlation between blood glucose and distance run in the exercise endurance protocol. These blood glucose data suggest that the GHSR-expressing MBH neurons direct glucose utilization as a fuel source to help mice run farther. In mice with inhibited GHSR-expressing MBH neurons, glucose is not efficiently utilized, leading to higher blood glucose levels and potentially decreased endurance. Further research, including more in-depth examination of lactate and glucose kinetics following exercise, is needed to test these hypotheses. It also would be worthwhile for future studies to explore why blood lactate was lowered by CNO administration in the “misses,” in which the activity of GHSR-expressing neurons outside the MBH was impacted but not their exercise endurance.

Another clue regarding potential downstream mediators comes from the novel observation that approximately 63.3% of hM4Di-infected, GHSR-expressing VMHvl neurons contain nNOS. This subpopulation of GHSR-expressing neurons is part of the total population of GHSR-expressing MBH neurons, which, when chemogenetically inhibited, reduced food intake after HIIE, exercise endurance, and ghrelin-induced food intake. Important metabolic and behavioral effects of nNOS in the VMH have been demonstrated. For instance, nNOS-derived NO production in the VMH is stimulated by insulin-induced hypoglycemia, is required for glucose sensing by VMH glucose-inhibited neurons, and is necessary for the usual counter-regulatory response to hypoglycemia (50, 51). Most nNOS-expressing VMHvl neurons have been characterized as glutamatergic and ERα^+^ (35), and ERα^+^ VMHvl neurons play key roles in sensing glucose fluctuations and preventing severe hypoglycemia (52). Further, chemogenetic activation of ERα and melanocortin 4 receptor (MC4R) coexpressing VMHvl neurons markedly increases spontaneous locomotor activity in male mice, female mice with intact ovaries, and estrogen-depleted ovariectomized female mice, which otherwise are less active than intact females (29). It is unclear if the GHSR + nNOS coexpressing VMHvl neurons observed here overlap with these described ERα and MC4R coexpressing VMHvl neurons. Studies are needed to investigate the role of nNOS and ERα in ghrelin’s effects on food intake after HIIE and exercise endurance.

We would be remiss in not mentioning some caveats of the exercise protocols used here. Although the HIIE and exercise endurance protocols were preceded by 2 adaptation days in which the mice were familiarized to the treadmills (which included 5 minutes at rest on the treadmills and then exercising them for 5 minutes × 8–10 m/min and then for 5 minutes × 10–12 m/min), the mice did not otherwise undergo a preceding training period, unlike most human athletes. Also, the mice were coaxed to continue running on the treadmill with the assistance of an electric stimulus generated by a shock grid present at the treadmill base (HIIE and exercise endurance) and by manually tapping their tails using a soft nylon bottle brush (HIIE), which could be viewed as stress, pain, or fear inducing. These prompts likely overlap only in part with the various motivational aides at play in human athletes. Additionally, the exercise protocols reported here were performed during the daytime, when mice usually have low levels — although not zero levels — of spontaneous physical activity. Despite these caveats, it is reasonable to assume that the same neurocircuits likely would be engaged had the exercise protocols been possible to perform without a shock grid or bottle brush and had they been performed during the nighttime using trained mice. Studies that incorporate those additional elements into the HIIE and exercise endurance protocols could test such a hypothesis.

## Methods

### Mice.

*Ghsr-IRES-Cre* mice (containing 1 copy of the *Ghsr-IRES-Cre* allele, from the Zigman lab) (21), mice derived from crosses between *Ghsr-IRES-Cre* mice and ROSA26-YFP mice [B6.129X1-Gt(ROSA)26Sor^tm1(EYFP)Cos^/J] (The Jackson Laboratory; stock 006148) containing 1 copy of the *Ghsr-IRES-Cre* allele and 1 ROSA26-YFP transgene, NPY-hrGFP (gift from Joel Elmquist, University of Texas Southwestern; UTSW) (26), and C57BL/6N (Charles River Laboratories) mice were used in this study. All lines had been backcrossed more than 10 generations onto a C57BL/6N genetic background. All studies were performed using male mice housed at standard room temperature (22°C–24°C) under a 12-hour dark/12-hour light cycle with ad libitum access to water and standard chow diet (2916 Teklad Global 16% protein diet, Envigo), except as indicated. Diagrammatic representations of the experimental protocols were prepared using CorelDraw 11 software (Corel).

### Stereotaxic surgeries and viral injections.

Stereotaxic surgery was performed as previously described (21), with some modifications, on mice under ketamine (120 mg/kg BW)/xylazine (16 mg/kg BW) i.p. anesthesia or 1.5% isoflurane gas anesthesia while restrained in a Kopf stereotaxic apparatus. Following standard disinfection procedures, a small incision (~1.0 cm) was made into the skin overlying the skull, a small hole was drilled into the skull using a high-speed rotary micromotor (Foredom), and 200 nL of virus was injected into one side of the brain (coordinates below) over 10 minutes using a pulled glass micropipette connected to an air pressure injector system. A micromanipulator (Model S48 Stimulator, Grass Technologies) was used to control injection speed at 20 nL/min. After a 10-minute wait, the micropipette was slowly retracted, and the procedure was repeated on the contralateral side. The incision site was closed using a surgical suture. The mice were monitored on a warming pad until awake, after which they were singly housed and administered buprenorphine 1 mg/kg BW s.c. every 12 hours for 24 hours and carprofen 5 mg/kg BW s.c. daily for 3 days to relieve pain. Mice were allowed to recover for 3 weeks prior to behavioral/physiological testing. Cre-dependent AAV2-hSyn-DIO-hM4D(Gi)-mCherry virus (32) (hM4Di; catalog 44362; Addgene; titer: 2.3E13 GC/mL) was targeted bilaterally to the MBH by use of the following brain coordinates based on a mouse brain atlas (53): (distance from bregma: –1.40 mm; lateral from midline: ±0.20 mm; ventral from brain surface: –5.65 mm). These mice were used to generate the data in Figures 3, 4, and 7. In a separate cohort, AAV2-hSyn-mCherry virus (“non–Cre-dependent mCherry control virus”; catalog 114472; Addgene; titer: 1.8 × 10^13^ genome copies/mL), AAV2-hSyn-DIO-mCherry (“Cre-dependent mCherry control virus”; catalog 50469; Addgene; titer: 2.1 × 10^13^ genome copies/mL), or hM4Di were targeted slightly less ventrally to the following brain coordinates: (distance from bregma: –1.40 mm; lateral from midline: ±0.20 mm; ventral from brain surface: –5.60 mm). These mice were used to generate the data in Figures 5, 6, and 8. Illustrations of the stereotaxic injections into the MBH were made using CorelDraw 11 software, and are inspired by the mouse brain atlas (53).

### HIIE protocol.

The HIIE protocol involved running mice on motorized treadmills (Exer-6; Columbus Instruments) as previously described (14, 54) (Figure 3B). Mice were first familiarized to the treadmills for 2 days prior to the exercise bout (day 1: 5 minutes rest on the treadmill followed by running for 5 minutes at the speed of 8 m/min and then 5 minutes at 10 m/min; day 2: 5 minutes rest on the treadmill followed by running for 5 minutes at 10 m/min and for 5 minutes at 12 m/min). On day 3, mice were subjected to an HIIE bout, as follows. Food was removed from home cages at the start of the light cycle (7 am) for a duration of 6 hours. At the fourth hour of food restriction, mice were administered saline or CNO (0.3 mg/kg BW i.p.; catalog C0832; MilliporeSigma) in a crossover fashion. One hour following saline or CNO administration, mice were rested on the treadmill for 5 minutes. Immediately after the 5 minutes of rest, they were submitted to a 1-hour exercise bout consisting of 3 × 20-minute intervals (5 minutes at a speed of 12 m/min, followed by 10 minutes at 17 m/min, then 5 minutes at 22 m/min), without rest between intervals. Mice were coaxed to continue running on the treadmill by an electric stimulus (0.25 mA × 163 V and 1 Hz) generated by a shock grid present at the treadmill base and by manually tapping their tails using a soft nylon bottle brush, as needed. Notably, during the HIIE protocol, all mice received 1 (not more and not less) electric shock during each of the three 20-minute intervals when running at 22 m/min speed; rarely did we witness mice receiving an electric shock at the lower speeds. After completion of the HIIE bout, blood glucose and blood lactate concentrations were determined immediately from blood from tail snips using a Bayer Contour glucometer and Nova Biomedical Lactate Plus meter, respectively; mice and standard chow (Teklad Global Diet, 2916) were reintroduced into the home cages; and food intake was measured over the next 30 minutes, 1 hour, 2 hours, and 4 hours.

### Exercise endurance protocol.

Exercise endurance was tested by subjecting mice to a stepwise running paradigm as described (14) with minor modifications (Figure 4A). The mice were first acclimatized to the treadmill for 2 days, as described for the HIIE protocol. On the day of the experiment (day 3), food was removed from home cages 4 hours after the start of the light cycle (7 am) for a duration of 2 hours. Five hours after the start of the light cycle (12 pm), mice were injected with either saline or CNO (0.3 mg/kg BW i.p.). One hour after the injections, mice were placed on the treadmill for 5 minutes at rest, followed by running with a starting speed of 10 m/min for 40 minutes, next by running at speeds that were increased at the rate of 1 m/min every 10 minutes until the speed reached 13 m/min, and finally by running at speeds that were increased at the rate of 1 m/min every 5 minutes until exhaustion. The exhaustion time was noted as the time at which the mice stopped running and remained on the electric shock grid for more than 5 seconds, without attempting to resume running (14, 19). Bottle brushes were not used to coax the mice to run. Just after exhaustion, mice were removed from the treadmill, and blood was collected from tail snips to measure blood glucose, blood lactate, and ghrelin.

Notably, although we did not measure hormone levels following the exercise endurance protocol in the current study, in our prior study using this same exercise endurance protocol (14), marked, genotype-independent rises in plasma corticosterone were noted at the point of exhaustion in both wild-type mice and GHSR-null littermates, the latter of which exhibited decreased running distance. Further, there was an effect of exercise to raise plasma norepinephrine but not plasma epinephrine when measured at the point of exhaustion, although exhausted GHSR-null mice had significantly lower epinephrine and norepinephrine levels than exhausted wild-type mice (14). Also, when wild-type mice were time-matched to run only as long as GHSR-null mice (to the point of exhaustion of the GHSR-null mice), epinephrine and norepinephrine levels were found to be significantly lower in the exhausted GHSR-null mice (14).

### Ghrelin-induced food intake studies.

Ghrelin-induced food intake was performed as before (21) (Figure 7A) and as described in the Supplemental Methods.

### oGTT.

oGTT was performed as before (39) (Figure 8B) and as described in the Supplemental Methods.

### Determination of plasma ghrelin and LEAP2.

Tail vein blood was collected and processed as described in the Supplemental Methods.

### RNAscope ISHH for Ghsr.

NPY-GFP (Figure 1, G–O), C57BL/6N (Figure 2, B–G, and Supplemental Figure 2), and mice carrying both a *Ghsr*-*IRES-Cre* allele and a ROSA26-YFP transgene (Figure 1, A–F, and Supplemental Figure 1, A–N) were deeply anesthetized with chloral hydrate (500 mg/kg BW i.p.) and perfused transcardially with diethyl pyrocarbonate–treated (DEPC-treated) 0.9% phosphate-buffered saline (PBS) followed by 10% neutral buffered formalin, using high-precision multichannel pump (Ismatec) (45, 55). Brains were removed and postfixed in 10% formalin overnight at 4°C and then cryoprotected in 25% sucrose solution in DEPC-treated PBS overnight at 4°C. After embedding in Tissue-Tek OCT compound, serial 25 μm–thick coronal sections extending from the olfactory bulb to the cervical spinal cord were obtained using a cryostat (Leica), immersed in DEPC-treated PBS buffer, and separated into 5 equal brain series. One series of hypothalamic sections containing the ARC, extending from –1.34 mm to –3.08 mm past bregma, was rinsed in DEPC-PBS, treated with 0.9% hydrogen peroxide for 10 minutes at room temperature, rinsed, mounted onto SuperFrost slides (Thermo Fisher Scientific), and dried overnight in a vacuum oven at 37°C. The next day, RNAscope ISHH was performed using the RNAscope Multiplex Fluorescent Kit v2 assay (Advanced Cell Diagnostics, ACD), as per the manufacturer’s instructions: slides were rinsed 2 times in PBS and placed in an oven for 30 minutes at 60°C. Afterward, slides were postfixed in 10% formalin for 15 minutes at 4°C, then gradually dehydrated in ethanol (50%, 70%, and 100%; 5 minutes each) before target retrieval for 15 minutes at 98°C–102°C. Slides were incubated in protease III (322337, ACD) for 30 minutes at 40°C, then washed in distilled water and incubated in RNAscope probes for *Ghsr* (Mm-GHSR; 426141, ACD) for 2 hours at 40°C. Sections were further processed using the RNAscope Multiplex Fluorescent Detection Reagents v2 kit (323110, ACD) using instructions provided by the manufacturer. Slides were immediately coverslipped using EcoMount medium (Biocare).

### Tissue processing for immunofluorescence.

Mice were deeply anesthetized with chloral hydrate (500 mg/kg BW i.p.) and transcardially perfused with 0.9% PBS followed by 10% neutral buffered formalin, using a high-precision multichannel pump, as described (45, 55) with some modifications. Brains were dissected, postfixed in the same fixative overnight at 4°C, and then cryoprotected by immersing in 25% sucrose solution in PBS overnight at 4°C. After embedding in Tissue-Tek OCT compound, serial 25 μm–thick coronal sections extending from the olfactory bulb to the cervical spinal cord were obtained using a cryostat, immersed in antifreeze solution, separated into 5 equal brain series, and then stored at –20°C until further processing. One series of hypothalamic sections containing the ARC, extending from –1.34 mm to –2.80 mm past bregma, was washed with PBS and then mounted on SuperFrost slides; dried overnight; and coverslipped with VECTASHIELD mounting medium with DAPI (catalog H-1200, Vector Laboratories). mCherry fluorescence was determined at the end of the study to classify those cases with appropriately targeted virus injections as “hits” or mistargeted virus injections as “misses” (Results).

Separate series of free-floating coronal sections — 1 series containing the sections approximately –1.82, –2.06 mm (Figure 5, C, D, F, and G, and Figure 7, D–G), and –2.30 mm (Figure 5, E and H) past bregma and another series containing a section approximately –1.94 mm past bregma (Figure 6) — were processed for c-Fos and nNOS, respectively, as described (45), with minor modifications. Sections were rinsed in PBS, immersed in 0.5% Triton X-100 solution in PBS for 30 minutes, and blocked in 3% normal donkey serum (catalog 017-000-121, Jackson ImmunoResearch Laboratories) in PBS for 2 hours. Sections were incubated either in diluted rabbit anti–c-Fos antibody (catalog ab190289; Abcam; dilution 1:1,000) or diluted rabbit anti-nNOS antibody (catalog 61-7000; Invitrogen; dilution 1:1,000) for 24 hours at room temperature. After washing in PBS, the sections were incubated in Alexa Fluor 488 donkey anti-rabbit IgG (catalog A21206, Invitrogen; dilution 1:500) for 2 hours at room temperature, and following additional washings in PBS, sections were mounted onto SuperFrost slides, dried overnight, and coverslipped with VECTASHIELD mounting medium with DAPI.

Separate series of free-floating coronal sections ranging from approximately –1.34 to –3.08 mm (Figure 1, A–F) past bregma from mice carrying 1 *Ghsr-IRES-Cre* allele and 1 ROSA26-YFP transgene underwent immunohistochemistry for YFP. The same protocol as described above was followed except that the primary antibody used was chicken anti-GFP antibody (catalog GFP-1010; Aves Labs, dilution 1:1,000), and the secondary antibody used was Alexa Fluor 488 goat anti-chicken IgG (catalog A11039, Invitrogen; dilution 1:500).

### Dual-label histochemistry.

Series of free-floating coronal sections ranging from approximately –1.70 to –2.06 mm past bregma from 5 mice carrying 1 *Ghsr-IRES-Cre* allele and 1 ROSA26-YFP transgene first underwent immunohistochemistry for YFP (Supplemental Figure 1) using RNase-free conditions. Sections were rinsed in DEPC-PBS and immersed in 0.5% Triton X-100 solution in DEPC-PBS containing 1 μL RNase inhibitor (catalog 100000840; RNaseOUT Recombinant Ribonuclease Inhibitor; Invitrogen) per 100 μL DEPC-PBS for 30 minutes. Sections were incubated in diluted chicken anti-GFP antibody (Aves Labs; dilution 1:1,000 + 1 μL RNase inhibitor per 100 μL DEPC-PBS) for 6 hours at room temperature. After washing in DEPC-PBS, the sections were incubated in Alexa Fluor 488 goat anti-chicken IgG (Invitrogen; dilution 1:500 + 1 μL RNase inhibitor per 100 μL DEPC-PBS) for 1 hour at room temperature. Following additional washings in RNase inhibitor-treated DEPC-PBS, sections were mounted onto SuperFrost slides and processed for RNAscope in situ hybridization histochemistry as described above.

### Image capture and analysis.

Most images were captured with either 10× or 20× objectives of a fluorescence microscope (Leica DM6 B digital research microscope with Leica DFC 9000 GT digital microscopy camera) plus LAS X software or a laser scanning confocal microscope (Zeiss LSM 880 airyscan) plus ZEN blue software. Single-, double-, or triple-labeled fluorescence images or laser-scanning images were captured using suitable filter sets or respective lasers for Alexa Fluor 488 (green), Alexa Fluor 594/mCherry (red), and DAPI (blue). Serial brain sections were captured with the fluorescence microscope and processed using LAS X software (Figure 1; Figure 2, B–G; Figure 3, C–H; Figure 7, D–G; and Supplemental Figures 1–4). A series of optical slices through the MBH was captured and processed using Zeiss ZEN blue image browsing software (Figure 5, C–H; Figure 6; Figure 8, C–E; and Supplemental Figure 5). Neuroanatomical colocalization between cells containing mCherry fluorescence (red) and c-Fos or nNOS immunoreactivity (green) appeared as an overlap of these 2 fluorophores (yellow). DAPI counterstaining (to identify nuclei) and comparisons to a mouse brain atlas (53) were used to identify the regional boundaries between different MBH regions. Cells showing clear, round, DAPI-stained nuclei surrounded by mCherry were identified as being mCherry positive. c-Fos–immunoreactive cells were identified by the presence of green nuclei with DAPI counterstaining. Cells showing clear, round, DAPI-stained nuclei surrounded by green fluorescence were identified as nNOS-immunoreactive cells. Manual counting of cells was performed with the assistance of the Adobe Photoshop 22.3.0 counting tool bilaterally for each mouse. See above for the levels of the ARC that were analyzed for c-Fos and nNOS. For PMV, 1 level approximately –2.30 mm past bregma was analyzed. Similarly, the colocalization of mCherry with c-Fos in the VMH, or mCherry with nNOS in the VMHvl, was assessed in each animal, and percentages were determined. The size and brightness of all the captured photomicrographs were adjusted uniformly with Adobe Photoshop.

To compare *Ghsr* mRNA expression between sedentary and HIIE-exposed C57BL/6N mice, images of coronal MBH sections from those mice were stained for *Ghsr* mRNA by RNAscope (red) and DAPI counterstaining (blue; to identify nuclei) and then were compared to a mouse brain atlas (53) to identify the boundaries between different MBH and nearby regions. No adjustments to intensity or exposure were made to the images. Boundaries for the ARC, VMH, and DMH were determined for each individual section and manually drawn on the images using the freehand tool in Adobe Photoshop. The ARC and VMH were analyzed bilaterally at 3 distances from bregma (–1.34 mm, –1.82 mm, and –2.06 mm), while the DMH was analyzed bilaterally at 2 distances from bregma (–1.82 mm and –2.06 mm), using ImageJ software (NIH; https://imagej.nih.gov/ij/): images were exported into ImageJ and converted to 8-bit grayscale to create a monochromatic image. A threshold for the red channel was set between 24 (min) and 100 (max). Afterward, with the assistance of the ImageJ Analyze and Measure tools, the following parameters were determined: area, area faction, integrated density, and minimum + maximum gray values. The percentage fluorescent area of *Ghsr* expression on each side of the ARC was determined for each of the 3 coronal levels described above and then averaged together for each animal. The same was done for the VMH (at 3 levels) and DMH (at 2 levels). The data were averaged for the sedentary mice (*n* = 4) and separately, for the HIIE-exposed mice (*n* = 5).

### Statistics.

Data are presented as mean ± SEM and are analyzed by paired Student’s *t* test (2 tailed), unpaired Student’s *t* test (2 tailed), 1-way ANOVA, or 2-way ANOVA, as indicated in the figure legends. Holm-Šidák post hoc testing was used to further investigate differences if significant ANOVA effects were found. Correlations were done by Pearson’s correlation and simple linear regression analysis. All data were analyzed using Prism version 9.0.2 (GraphPad Software). No outliers were detected by Grubb’s test. *P* < 0.05 was considered statistically significant.

### Study approval.

All experiments were approved by the Institutional Animal Care and Use Committee of UTSW Medical Center.

### Data availability.

Values for all data points shown in graphs and behind any reported means are available in the Supporting Data Values.

## Author contributions

OS and JMZ developed the concept; OS and JMZ developed the experimental strategy. OS, SBO, SV, KS, DG, and SP performed the experiments. SOL worked with CPR, NPM, and CL to organize the breeding schedule and all aspects of animal husbandry required for the study. OS and JMZ analyzed the data. JMZ secured funding for the project. LLM helped edit the paper and troubleshoot experimental protocols. OS and JMZ wrote the paper.

## Supplementary Material

Supplemental data

Supporting data values

## Figures and Tables

**Table 1:**
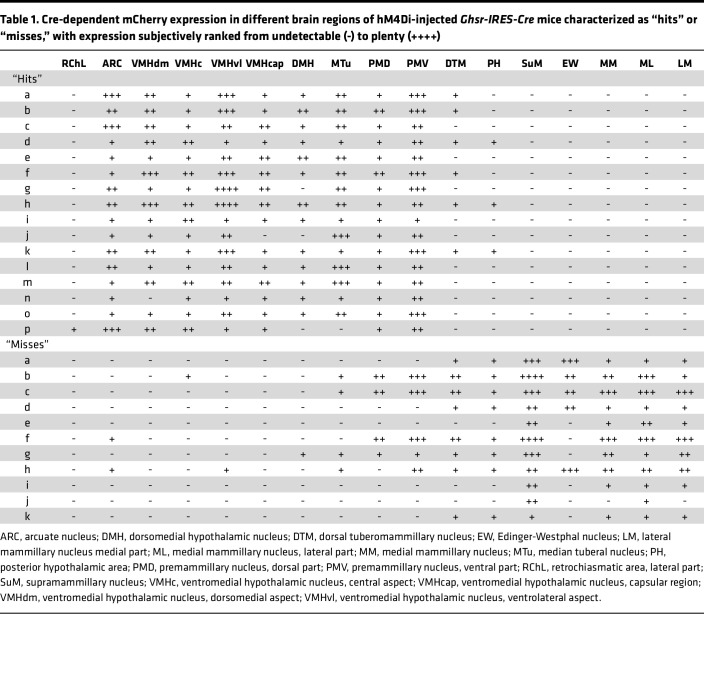
Cre-dependent mCherry expression in different brain regions of hM4Di-injected *Ghsr-IRES-Cre* mice characterized as “hits” or “misses,” with expression subjectively ranked from undetectable (-) to plenty (++++)

## Version 1 11/14/2023

In-Press Preview

## Version 2 12/22/2023

Electronic publication
