# Supplementary material for: Ghrelin-responsive mediobasal hypothalamic neurons mediate exercise-associated food intake and exercise endurance
Source: JCI Insight. 2023 Dec 22;8(24):e172549. doi: 10.1172/jci.insight.172549 (PMC10807726; doi:10.1172/jci.insight.172549)
Supplement: Supplemental data [file jciinsight-8-172549-s045.pdf]

Supplemental Methods

Ghrelin induced food intake studies

Mice were first handled for 3 days to acclimate them to the handling needed at the time of injections. Food was withdrawn from the cages during the light cycle (at ~10 AM) for 2 h, after which CNO (0.3 mg/kg BW i.p.) was delivered to all of the mice. One hour after CNO administration, the mice were administered ghrelin (1 mg/kg BW s.c., SP-GHRL-1, Innovagen, Lund, Sweden), and standard chow (Teklad Global Diet [2916]) was re-introduced into the cage. Food intake was measured at 2 h post-ghrelin administration and mice were immediately anesthetized with chloral hydrate (500 mg/kg BW i.p.) and transcardially perfused.

Oral glucose tolerance test (oGTT)

Food was removed from the home cage 3 h after the start of the light cycle for a 6 h duration. During the 5th hour of fasting (at 3 PM), mice were administered CNO (0.3 mg/kg BW i.p.) to inhibit the GHSR-expressing neurons infected with hM4Di. After 1h, D-glucose (2 mg/kg BW; Sigma-Aldrich) was administered by oral gavage and blood glucose levels were determined at 0, 30, 60, 90 and 120 min from blood obtained from tail snips, as above.

Determination of plasma ghrelin and LEAP2

Tail vein blood was collected into two separate EDTA-coated Microvette® CB300 collection tubes on ice. The tubes contained one of the following protease inhibitors: p-hydroxymercuribenzoic acid (Sigma-Aldrich-; final concentration 1 mM; for ghrelin measurement) or aprotinin (Sigma-Aldrich; final concentration 250 KIU/mL; for LEAP2 measurement). The samples were immediately centrifuged at 4°C at 1,500 g x 15 min. For ghrelin stabilization, HCl was added (final concentration 0.1 N). Ghrelin concentrations were determined with an acyl-ghrelin ELISA kit (EMD Millipore, Billerica, MD; Catalog #EZRGRA-90K) after plasma samples were diluted 4X in assay buffer. LEAP2 concentrations were determined with a LEAP2 (37-76) (Mouse) ELISA kit (Phoenix Pharmaceuticals, Inc., Burlingame, CA, Catalog # EK-075-40) after plasma samples were diluted 4X in assay buffer. Endpoint calorimetric measurements were performed using a BioTek PowerWave XS Microplate spectrophotometer and KC4 junior software (Winooski, VT).

Supplemental Figures

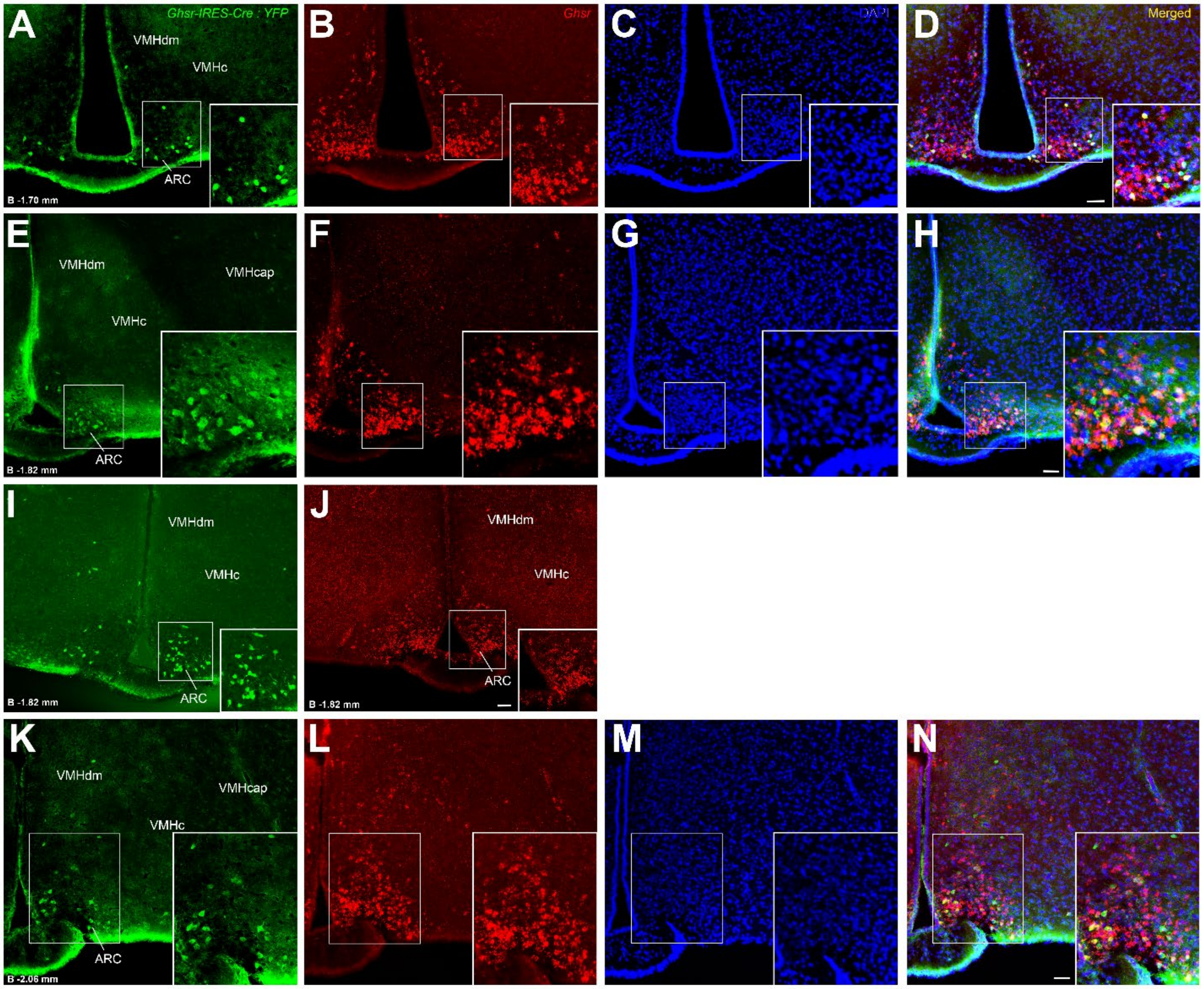

Supplemental Figure 1

Supplemental Figure 1. Further validation of the expected Cre recombinase activity pattern within *Ghsr-IRES-Cre* mice X ROSA26-YFP reporter mice. A-H and **K-N,** Low magnification fluorescence photomicrographs of two representative mice (**A-D**, "mouse 1"; **E-H** and **K-N**, "mouse 2") showing YFP-immunoreactive cell bodies (green) and *Ghsr* mRNA expression using RNAscope *in situ* hybridization histochemistry (red) and their co-localization (yellow) in coronal MBH sections (a total of n=5 mice were assessed, but only 2 are shown here). **I-J,** Low magnification fluorescence photomicrographs showing YFP-immunoreactive cell bodies (green) and *Ghsr* mRNA expression (red) in adjacent coronal MBH sections of "mouse 2" (the sections in **I** and **J** are adjacent to each other and to the section depicted in **E-H**). Scale bars = 50 µm in **A-N**. Approximate distance of each coronal section from bregma (B) is indicated in the lower left corner of each panel. ARC - Arcuate nucleus; VMHc - Ventromedial hypothalamic nucleus, central aspect; VMHcap - Ventromedial hypothalamic nucleus, capsular region; VMHdm - ventromedial hypothalamic nucleus, dorsomedial aspect.

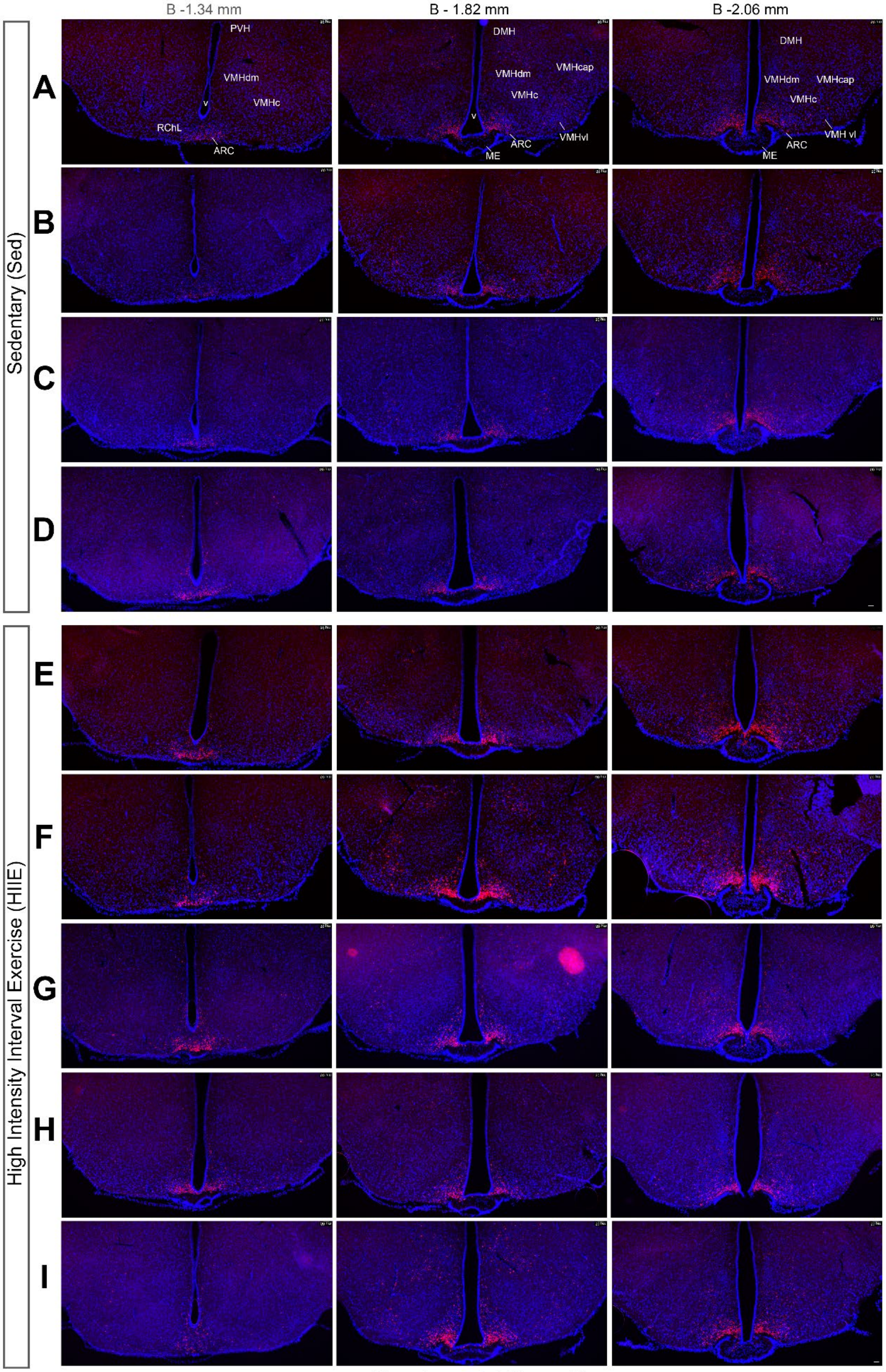

Supplemental Figure 2

Supplemental Figure 2. Hypothalamic *Ghsr* mRNA expression in sedentary C57BL/6N mice vs. C57BL/6N mice exposed to HIIE. A-I, Low magnification fluorescence photomicrographs showing *Ghsr* mRNA expression (red), as determined using RNAscope *in situ* hybridization histochemistry in coronal MBH sections of sedentary mice (**A-D**) and HIIE-exposed mice (**E-I**). DAPI (blue) is used as counterstaining. The approximate distances of each coronal section from bregma (B) are indicated at the top of each panel. Scale bars = 50 µm in **A-I**. v - third ventricle; ARC - Arcuate nucleus; DMH - Dorsomedial hypothalamic nucleus; ME-median eminence; PVH - Paraventricular hypothalamic nucleus; RChL - Retrochiasmatic area, lateral part; VMHc - Ventromedial hypothalamic nucleus, central aspect; VMHcap - Ventromedial hypothalamic nucleus, capsular region; VMHdm - ventromedial hypothalamic nucleus, dorsomedial aspect; VMHvl - Ventromedial hypothalamic nucleus, ventrolateral aspect.

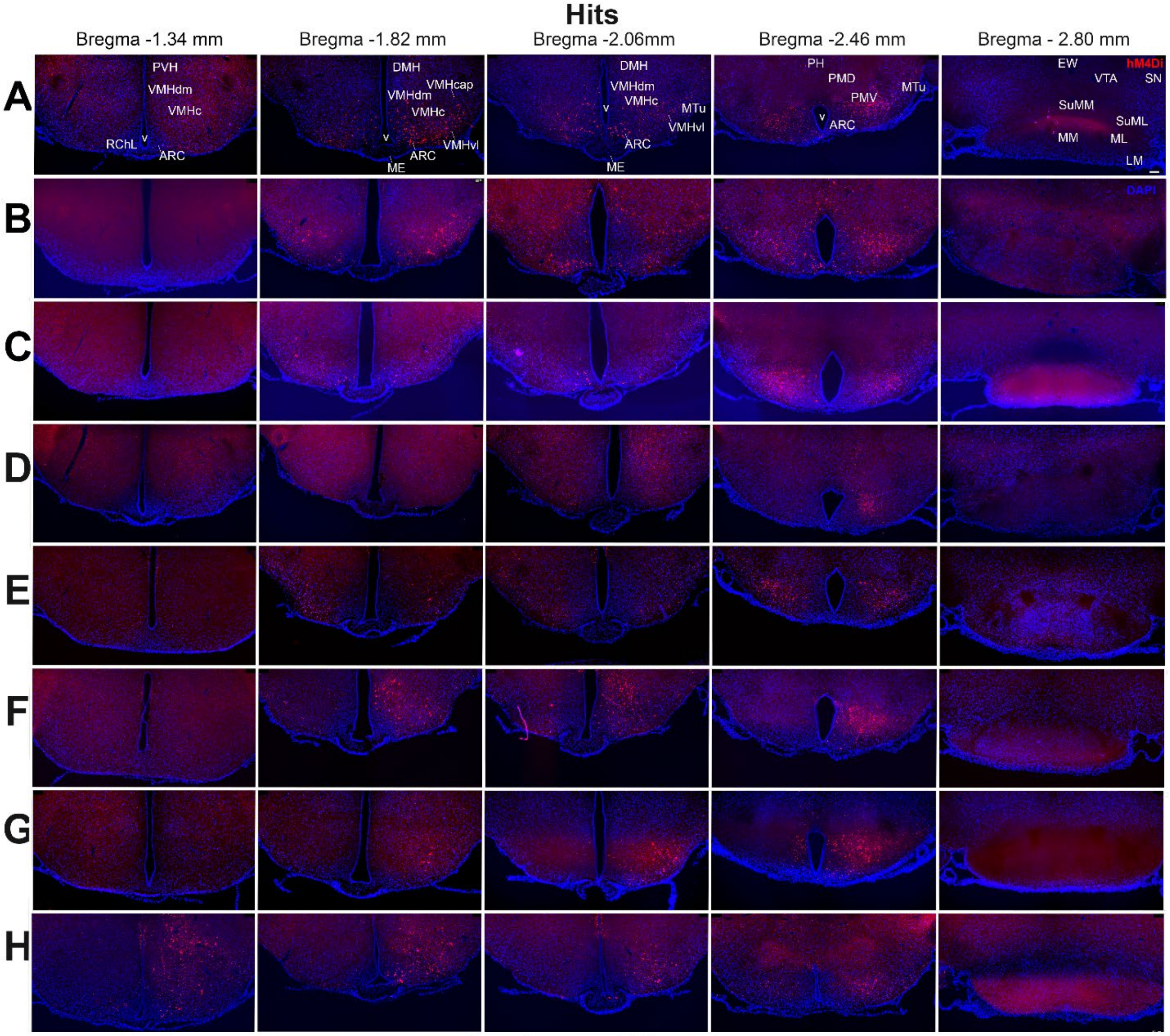

Supplemental Figure 3

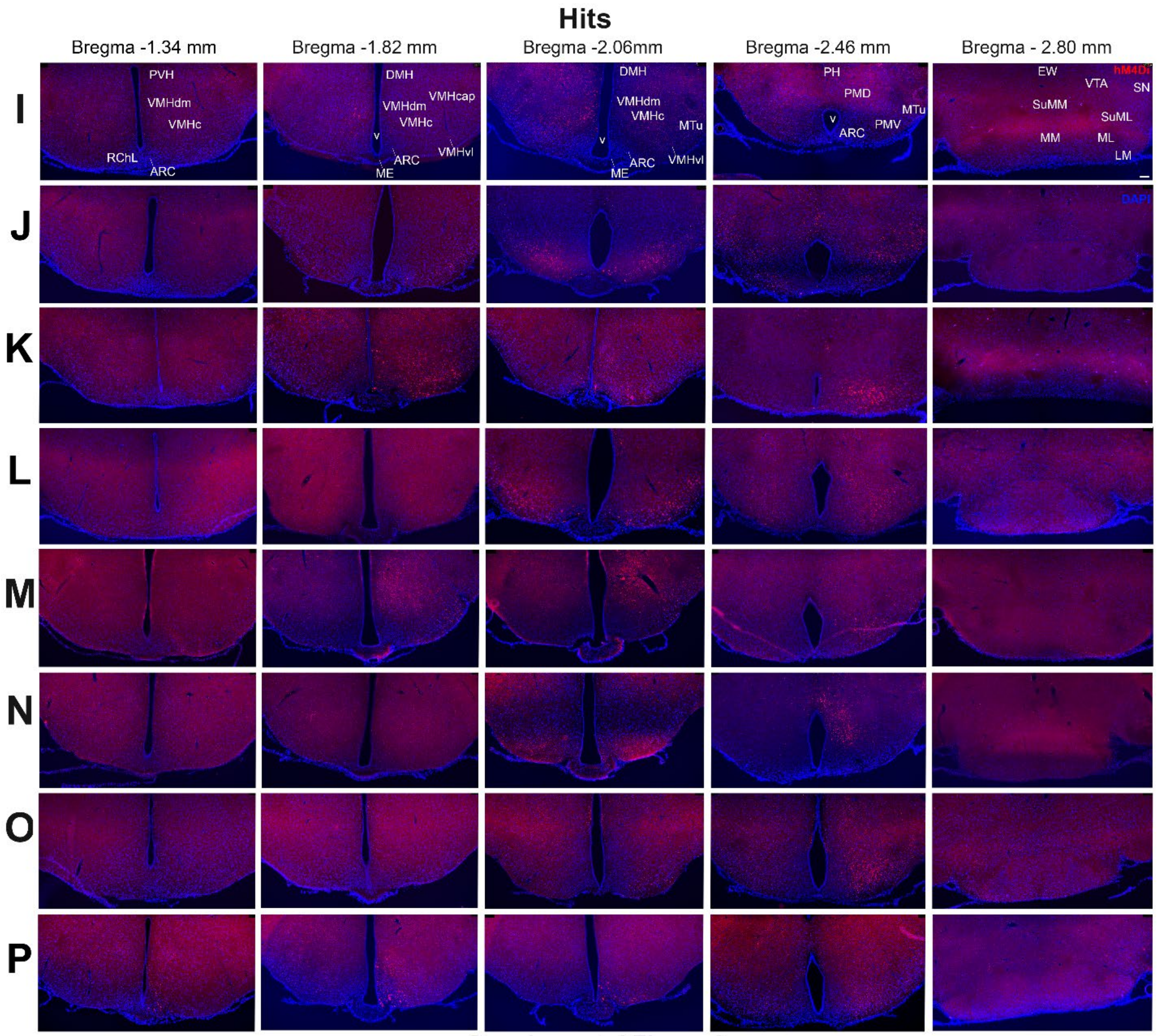

Supplemental Figure 3

Supplemental Figure 3. mCherry expression in AAV-hSyn-DIO-hM4D(Gi)-mCherry virus (hM4Di)-injected *Ghsr-IRES-Cre* "hits". Fluorescence photomicrographs showing Cre-dependent mCherry expression (red) in GHSR-expressing neurons within five coronal brain sections extending from approximately -1.34 mm to -2.80 mm from Bregma. Each of the n=16 "hits" is represented by a row (**A-P**). Qualitative assessment of the regions with expression and the amounts of expression for each "hit" is also included in Table 1. Scale bar in row **A** = 100 µm and applies to all the panels in rows **A**-**P**. DAPI counterstaining is shown in blue. v - third ventricle; ARC - Arcuate nucleus; DMH - Dorsomedial hypothalamic nucleus; EW - Edinger-Westphal nucleus; LM - lateral mammillary nucleus; ME- median eminence; MM - medial mammillary nucleus, medial part; ML - medial mammillary nucleus, lateral part; MTu - median tuberal nucleus; PH - posterior hypothalamic area; PMD - Premammillary nucleus, dorsal part; PMV -Premammillary nucleus, ventral part; PVH - Paraventricular hypothalamic nucleus; RChL - Retrochiasmatic area, lateral part; SN - Substantia nigra; SuML - Supramammillary nucleus, lateral part; SuMM - Supramammillary nucleus, medial part; VMHc - Ventromedial hypothalamic nucleus, central aspect; VMHcap - Ventromedial hypothalamic nucleus, capsular region; VMHdm - ventromedial hypothalamic nucleus, dorsomedial aspect; VMHvl - Ventromedial hypothalamic nucleus, ventrolateral aspect; VTA - Ventral tegmental area.

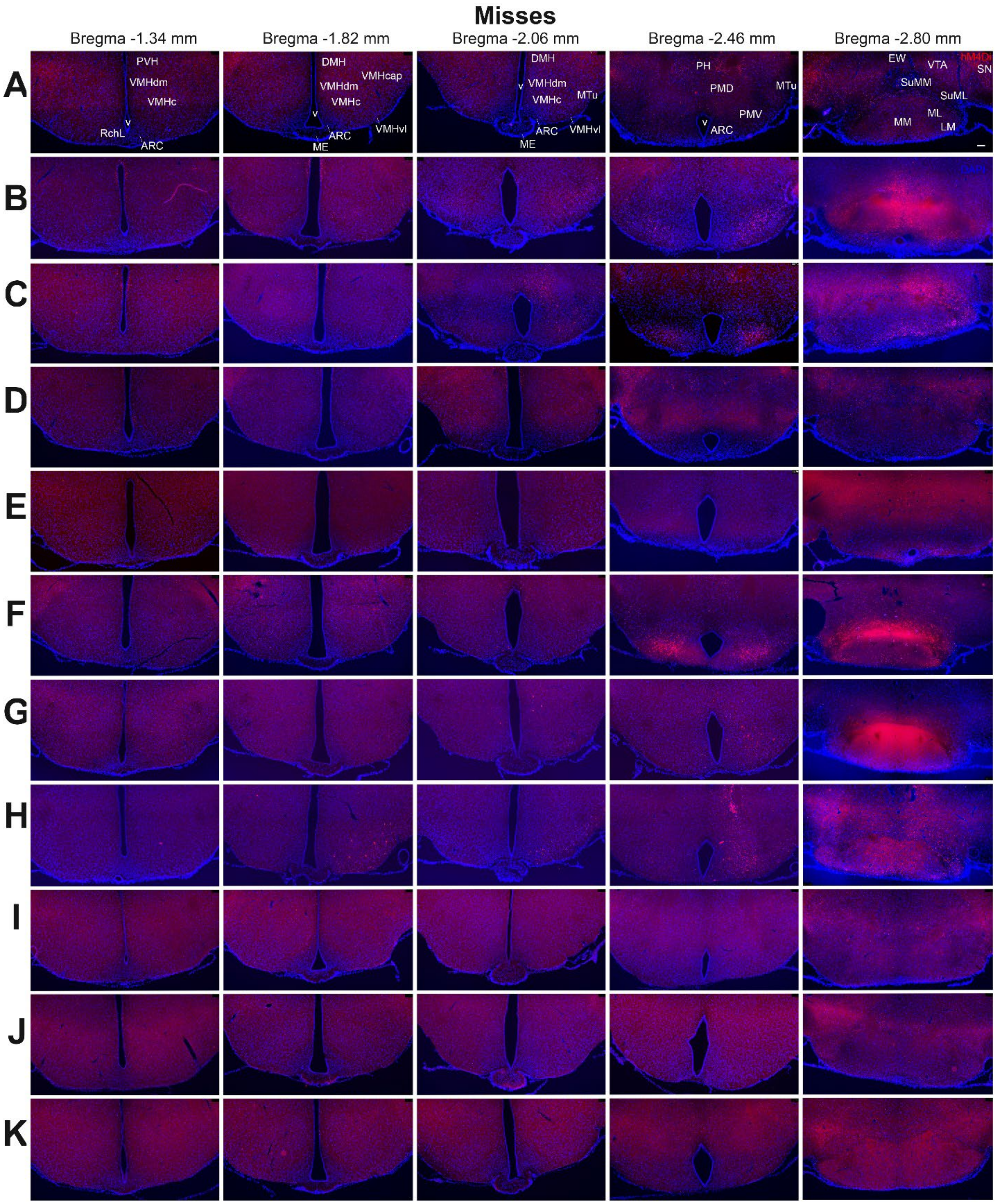

Supplemental Figure 4

Supplemental Figure 4. mCherry expression in AAV-hSyn-DIO-hM4D(Gi)-mCherry virus (hM4Di)-injected *Ghsr-IRES-Cre* "misses". Fluorescence photomicrographs showing Cre-dependent mCherry expression (red) in GHSR-expressing neurons within five coronal brain sections extending from approximately -1.34 mm to -2.80 mm from Bregma. Each of the n=11 "misses" is represented by a row (**A**-**K**). Qualitative assessment of the regions with expression and the amounts of expression for each "hit" is also included in Table 1. Scale bar in row **A** = 100 µm and applies to all the panels in rows **A**-**K**. DAPI counterstaining is shown in blue. v - third ventricle; ARC - Arcuate nucleus; DMH - Dorsomedial hypothalamic nucleus; EW - Edinger-Westphal nucleus; LM - lateral mammillary nucleus; ME- median eminence; MM - medial mammillary nucleus, medial part; ML - medial mammillary nucleus, lateral part; MTu - median tuberal nucleus; PH - posterior hypothalamic area; PMD - Premammillary nucleus, dorsal part; PMV -Premammillary nucleus, ventral part; PVH - Paraventricular hypothalamic nucleus; RChL - Retrochiasmatic area, lateral part; SN - Substantia nigra; SuML - Supramammillary nucleus, lateral part; SuMM - Supramammillary nucleus, medial part; VMHc - Ventromedial hypothalamic nucleus, central aspect; VMHcap - Ventromedial hypothalamic nucleus, capsular region; VMHdm - ventromedial hypothalamic nucleus, dorsomedial aspect; VMHvl - Ventromedial hypothalamic nucleus, ventrolateral aspect; VTA - Ventral tegmental area.

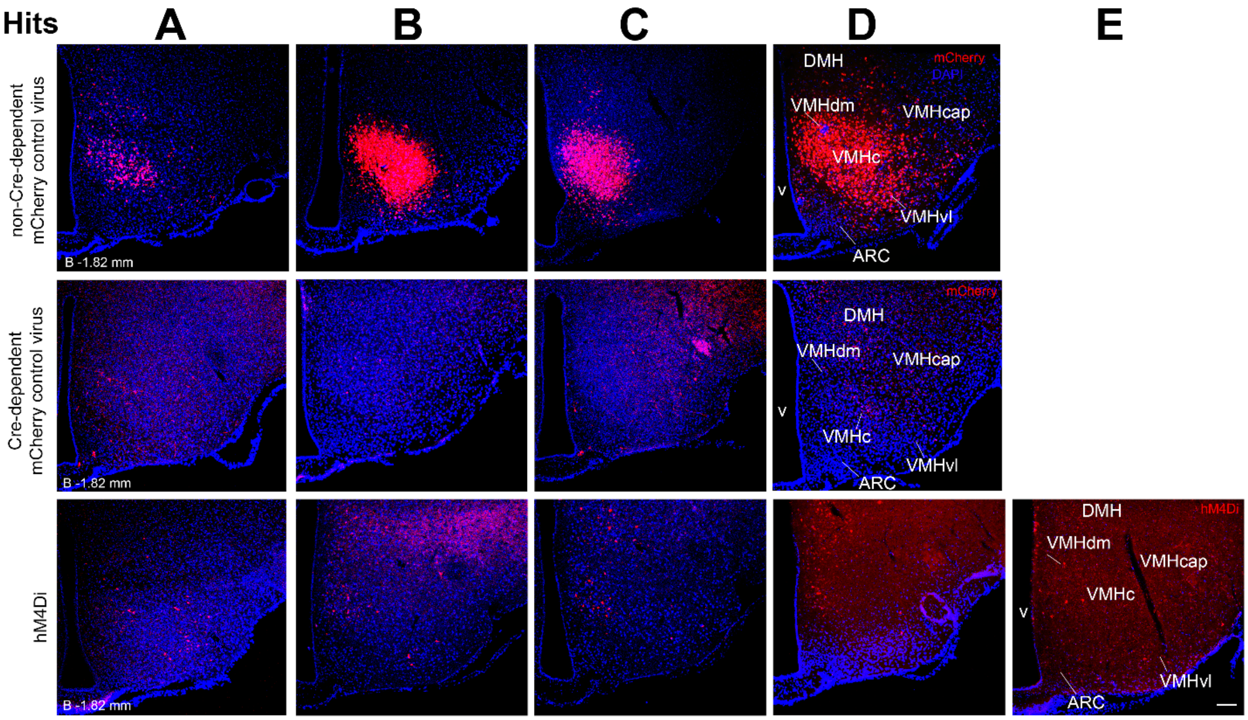

Supplemental Figure 5

Supplemental Figure 5. mCherry expression in the MBH of representative *Ghsr-IRES-Cre* "hits" injected with hM4Di or one of two control viruses and used to generate data in Fig. 5. Confocal photomicrographs showing mCherry expression (red) within the MBH of coronal sessions located approximately -1.82 mm bast Bregma (B) from **top row**, each of four (**A-D**) "hits" targeted with "non-Cre-dependent mCherry control virus" (AAV-hSyn-mCherry), **middle row,** each of four (**A-D**) "hits" targeted with "Cre-dependent mCherry control virus" (AAV-hSyn-DIO-mCherry), and **bottom row,** each of five (**A-E**) "hits" targeted with hM4Di (AAV-hSyn-DIO-hM4D(Gi)-mCherry). Scale bar in **E** = 100 μm and applies to all the panels. v - third ventricle; ARC – Arcuate nucleus; VMHc - Ventromedial hypothalamic nucleus, central aspect; VMHcap - Ventromedial hypothalamic nucleus, capsular region; VMHdm - ventromedial hypothalamic nucleus, dorsomedial aspect; VMHvl - Ventromedial hypothalamic nucleus, ventrolateral aspect.

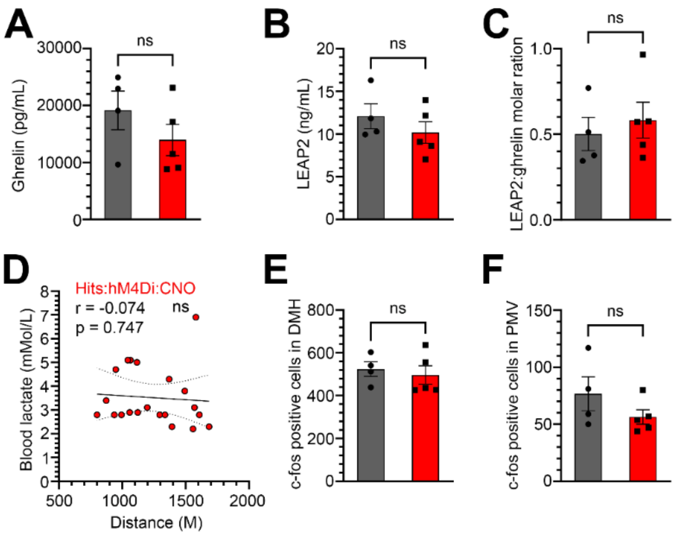

Supplemental Figure 6

Supplemental Figure 6. Additional effects of inhibiting GHSR-expressing MBH neurons in mice submitted to an exercise endurance test. Graphs showing effects of administration of CNO (0.3 mg/kg BW, i.p.) in hM4Di "hits" (n=5) vs. Cre-dependent mCherry control "hits" (n =4) on **A**, plasma ghrelin, **B**, plasma LEAP2, and **C**, plasma LEAP2:ghrelin molar ratio in exercised mice at exhaustion. **D,** Correlations between blood lactate with distance run during the exercise endurance test in CNO-treated hM4Di "hits" (n = 21, which includes the 5 hM4Di "hits" from this study and the 16 "hits" from Fig. 4). **E-F,** Numbers of c-fos-immunoreactive cells in the **E,** DMH and **F,** PMV of hM4Di "hits" (n=5) vs. Cre-dependent mCherry control "hits" (n =4) following CNO and the exercise endurance test. Data were analyzed by unpaired Student's t test (**A-C**, **E-F**) or by Pearson's correlation and simple linear regression analysis (**D**), whereby Pearson's correlation coefficient (r) and *p* values are indicated in the figure panels and whereby the solid lines represent the fitted linear regression curves and the dotted lines represent the s.e.m. ns - no statistically significant difference.
